# Spatial and seasonal patterns of tick distribution and climate-driven shifts in Jiangsu Province, an *SFTS*-affected region of eastern China

**DOI:** 10.1371/journal.pntd.0014847

**Published:** 2026-11-04

**Authors:** Hongliang Chu, Zhiwei Cheng, Zhiming Wu, Yihan Lu, Ye Yao

**Affiliations:** 1 Department of Disinfection and Vector Control, Jiangsu Provincial Center for Disease Control and Prevention, Nanjing, Jiangsu, China; 2 Department of Epidemiology, School of Public Health, Fudan University, Shanghai, China; 3 Key Laboratory of Public Health Safety of Ministry of Education, Fudan University, Shanghai, China; 4 Department of Biostatistics, School of Public Health, Fudan University, Shanghai, China; Huadong Research Institute for Medicine and Biotechniques, CHINA

## Abstract

**Background:**

Ticks are important vectors of zoonotic pathogens and play a key role in the transmission of severe fever with thrombocytopenia syndrome (*SFTS*) in East Asia. However, a comprehensive understanding of their spatial distribution, seasonal activity, and climate-driven habitat suitability remains limited, particularly at the provincial scale.

**Methods:**

This study analyzed long-term surveillance data on free-living ticks in Jiangsu Province, an *SFTS*-affected region in eastern China, from 2019 ~ 2024. Seasonal dynamics and habitat-associated differences were characterized using descriptive statistics. Environmental determinants of tick distribution and projected climate-driven changes in habitat suitability were assessed using an optimized MaxEnt modeling framework.

**Results:**

A total of 8,860 ticks belonging to seven species across three genera were identified, with *Haemaphysalis longicornis* (89.1%) and *Haemaphysalis flava* (9.8%) as the dominant species. Tick density varied seasonally, with post-larval ticks predominating during the April peak and larvae predominating during the September peak. Habitat preference differed between dominant species: *H. longicornis* was most frequently recorded in rural villages (33.7%) and country parks (18.3%), whereas *H. flava* was predominantly found in urban parks (24.8%) and forest parks (20.0%). Elevation, maximum temperature of the warmest month (bio5), and precipitation of the driest month (bio14) were key environmental determinants. Under current climatic conditions, highly suitable habitats were concentrated in the southwestern hilly areas. Under the SSP245 scenario (SSP, Shared Socioeconomic Pathway), the area classified as highly suitable using scenario-specific Jenks natural breaks was larger in future projections. Compared with the current scenario-specific high-suitability area, this class increased by 336.5% and 640.3% for *H. longicornis* and by 91.4% and 118.8% for *H. flava* during 2021 ~ 2040 and 2041 ~ 2060, respectively.

**Conclusion:**

Tick distribution in eastern China is strongly shaped by climatic constraints and is projected to undergo spatial reorganization, with larger areas falling into the scenario-specific high-suitability class under future climate scenarios. These findings underscore the importance of integrating climate-sensitive vector surveillance into *SFTS* prevention strategies within a One Health framework.

## Author summary

Severe fever with thrombocytopenia syndrome is an emerging tick-borne disease in East Asia. Understanding where important tick species are likely to occur is essential for planning surveillance and prevention, especially as climate conditions continue to change. In this study, we analyzed six years of tick surveillance data from Jiangsu Province, China, and combined these field observations with ecological modeling to estimate suitable habitats for the two dominant tick species, *Haemaphysalis longicornis* and *Haemaphysalis flava*. We found that these ticks showed seasonal patterns and differed across habitats. Their suitable areas were also shaped by environmental conditions, including temperature, precipitation, and elevation. Under future climate scenarios, suitable habitats were projected to become more extensive and better connected across the province. Our findings identified where vector ticks may be more likely to persist or become established in new areas. This information can help public health agencies design climate-sensitive tick surveillance, prioritize monitoring areas, and prepare more adaptive prevention strategies within a One Health framework.

## Introduction

Ticks are major vectors of zoonotic pathogens and represent an increasing threat to human and animal health worldwide [1,2]. Their distribution, abundance, and seasonal activity are strongly shaped by environmental conditions, resulting in substantial spatial and temporal heterogeneity in exposure risk [3–6]. Understanding how climatic and landscape factors structure tick populations is therefore central to anticipating vector-borne disease risk under ongoing environmental change.

In East Asia, tick-borne diseases have gained increasing public health attention, particularly following the emergence of Severe Fever with Thrombocytopenia Syndrome (*SFTS*) [7]. Caused by *Bandavirus dabieense* [8], *SFTS* is characterized by an acute febrile illness with thrombocytopenia and leukocytopenia and is associated with a relatively high case-fatality rate [9,10]. Since its first identification in China [11], indigenous *SFTS* cases have been reported in Japan and South Korea [12–14], highlighting the regional significance of this emerging tick-borne disease. Because human infection occurs primarily through bites from infected ticks [15,16], characterizing tick ecology and distribution is essential for assessing *SFTS* transmission risk and informing prevention strategies within a One Health framework [17].

In China, SFTS cases have been documented in multiple provinces. However, the affected areas are predominantly concentrated in the central and eastern regions. This geographical clustering of cases is closely associated with the regional distribution of tick vectors [18–20]. Jiangsu Province, located along the lower reaches of the Yangtze River, is a recognized *SFTS*-affected area in eastern China and has reported sustained local transmission [21]. Between 2014 and 2023, a total of 914 *SFTS* cases were reported in Jiangsu Province, with an average annual incidence of 0.11 per 100,000 population and an overall case fatality rate of 7.44% [22]. Local tick and pathogen investigations in the region also point to persistent ecological risk. In Nanjing City, the capital of Jiangsu Province, a 2024 investigation found that free-living ticks were mainly *Haemaphysalis longicornis* (hereafter referred to as *H. longicornis*), accounting for 75.65%; and *Haemaphysalis flava* (hereafter referred to as *H. flava*) accounting for 23.91%. Pathogen testing showed a 25.49% positivity rate for Rickettsia in tick samples, and the *Bandavirus dabieense* seropositivity rate among animal hosts was 0.65% [23]. More recent city-level surveillance further showed a consistent dominance of *H. longicornis* and *H. flava* across Jiangsu Province: In Suzhou City, a study collected 148 ticks and found that *H. longicornis* (47.3%) was the predominant species, with spotted fever group *Rickettsiae* detected at a minimum infection rate of 2.8% [24]. Monitoring of free-living ticks in Yangzhou City from 2020 to 2024 detected only two tick species, *H. longicornis* and *H. flava*, accounting for 70.01% and 29.99% of collected ticks, respectively [25], and surveillance in Xuzhou City from 2020 to 2023 reported *H. longicornis* as the dominant free-living tick species (92.08%) [26]. Together, these findings suggest that *H. longicornis* is consistently the dominant free-living tick species in Jiangsu, *H. flava* is an important secondary species.

Previous experimental studies have demonstrated that both *H. longicornis* and *H. flava* can acquire *Bandavirus dabieense* during blood feeding and subsequently support viral maintenance and transmission [27,28]. Therefore, a systematic, province-wide assessment of their population dynamics, habitat associations, and spatial suitability is of substantial public-health importance for *SFTS* prevention and control in Jiangsu Province. Such evidence can help move local tick surveillance beyond fragmented city-level observations toward risk-informed, climate-sensitive vector monitoring and targeted interventions for the dominant tick species implicated in regional *SFTS* ecological risk.

Ecological niche models (ENMs), especially the maximum entropy model (MaxEnt), are widely used to project species distributions under current and future climate scenarios [29–31]. In China, Yang et al. [32] and Zhao et al. [33] used tick occurrence records from public datasets and published studies with the MaxEnt model to project the potential distributions of multiple tick species at the national scale. Ding et al. [34] further linked projected *H. longicornis* suitability with *SFTS* case data in a multi-model, multi-scenario framework, revealing spatially heterogeneous changes in vector suitability and *SFTS* incidence under climate change. In addition, the county-level tick and pathogen atlas developed by Zhao et al. [35] integrated records for 124 tick species, 103 tick-borne agents, and human infections associated with 29 pathogen species or subspecies, and showed that predicted suitable habitats for predominant vector ticks were substantially broader than documented occurrence areas. Together, these studies have provided important national-scale evidence on tick diversity, tick-borne pathogens, and climate-sensitive distribution risks, with clear implications for public health planning and policy. However, most existing maps and models have been developed at relatively broad spatial scales, often relying on compiled occurrence records, pathogen detections, or reported human cases. As a result, they may not fully capture the seasonal dynamics, habitat-specific patterns, and local ecological heterogeneity of free-living ticks within a single *SFTS*-affected province. Their direct transferability to tick surveillance and *SFTS* prevention in Jiangsu Province therefore remains limited.

To address this gap, we analyzed province-wide surveillance data on free-living ticks collected in Jiangsu Province from 2019 to 2024. We characterized the seasonal and habitat-specific distribution patterns of dominant tick species and estimated their potential spatial distributions under current and future climatic conditions using an optimized MaxEnt framework [36]. The novelty of this study lies not in the application of ecological niche modeling alone, but in the integration of multi-year, locally collected surveillance data with province-scale modeling in an *SFTS*-affected region of eastern China. By linking field surveillance evidence with climate projections for *H. longicornis* and *H. flava*, this study provides a locally calibrated and spatially explicit assessment of tick habitat suitability of the two dominant species that may inform climate-sensitive surveillance, targeted vector control, and *SFTS* prevention strategies in Jiangsu and comparable eastern Chinese settings.

## Results

### Characterization of tick distribution

Between 2019 and 2024, a total of 7 tick species, belonging to 3 genera, were identified through routine surveillance in 256 surveillance sites across Jiangsu Province (Fig 1). The species included *Haemaphysalis longicornis*, *Haemaphysalis flava*, *Haemaphysalis phasiana*, *Haemaphysalis campanulata*, *Haemaphysalis hystricis*, *Rhipicephalus microplus*, and *Ixodes sinensis* (Table 1). *H. longicornis* (89.1%) and *H. flava* (9.8%) were the dominant species. The remaining species were detected at only a limited number of sites.

**Table 1:** Tick species identified in Jiangsu Province, eastern China, 2019 ~ 2024.

Family	Genus	Species	Number	Surveillance events
**Ixodidae**	***Haemaphysalis***	*H. longicornis*	7897	404
		*H. flava*	868	123
		*H. phasiana*	90	18
		*H. campanulata*	1	2
		*H. hystricis*	1	1
	***Rhipicephalus***	*R. microplus*	2	1
	***Ixodes***	*I. sinensis*	1	1

**Fig 1:**
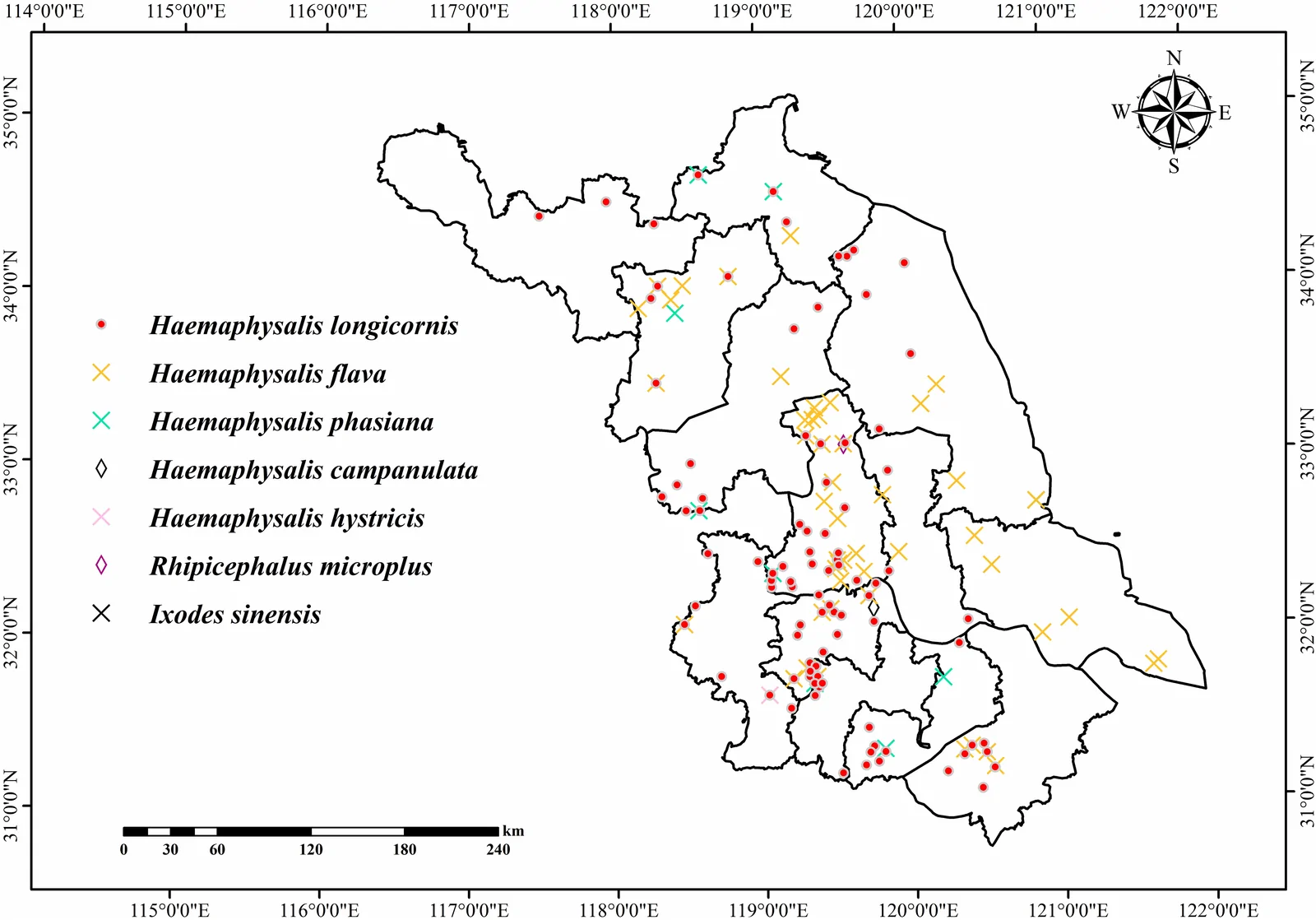
Spatial distribution of tick surveillance sites in Jiangsu Province, 2019 ~ 2024. The basic map is sourced from the Chinese Resource and Environmental Science Data Platform (http://www.resdc.cn/, https://doi.org/10.12078/2023010102).

For the two dominant tick species, the surveillance events were the most in April, May and September, and the tick density also fluctuated seasonally. Monthly total tick density showed seasonality in *H. longicornis* from 2019 to 2024 (Fig 2a), with the highest mean density in September (5.165 ticks/100 m), followed by April (4.062 ticks/100 m) and June (3.215 ticks/100 m). Larvae peaked in September, whereas post-larval stages peaked in April and May. Raw data were non-normal (W = 0.495, *P* < 0.001) but homoscedastic (F = 0.981, *P* = 0.444). One-way ANOVA was non-significant (F = 1.07, *P* = 0.385), whereas log-transformed analysis showed a significant monthly effect (F = 2.46, *P* = 0.017), with lower density in August than in April and September (Tukey, *P* < 0.05). Seasonal variation was also observed in *H. flava* (Fig 2b), with highest mean density in April (1.394 ticks/100 m), followed by September (1.111 ticks/100 m) and July (0.895 ticks/100 m). Larvae peaked in September, and post-larval ticks in April. Data were non-normal (W = 0.576, *P* < 0.001) but had equal variances (F = 0.567, *P* = 0.803). ANOVA showed no significant monthly differences in raw or log-transformed data (*P* > 0.7), and no significant pairwise comparisons were detected. Notably, monthly surveillance events were positively correlated with monthly total tick density (Pearson r = 0.785, *P* = 0.012; Spearman ρ = 0.883, *P* = 0.002), suggesting that sampling effort may have influenced the observed seasonal pattern to some extent.

**Fig 2:**
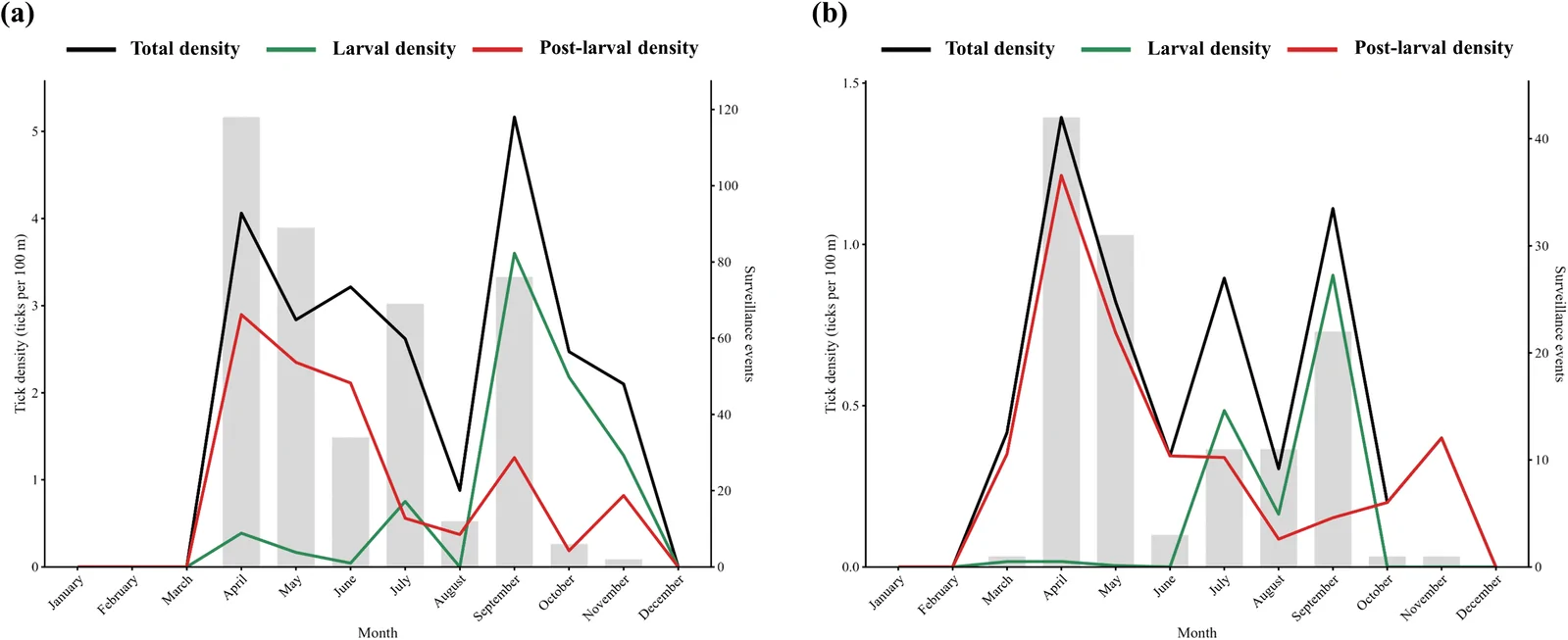
Seasonal dynamics of tick density and surveillance events in Jiangsu Province, 2019 ~ 2024. (a) *H. longicornis*; (b) *H. flava*. Monthly mean tick density is shown by lines, including total density (black), larval density (green), and post-larval density (red). Post-larval ticks comprise nymphs and adults. Light grey bars represent the number of surveillance events for each month and are plotted on the secondary y-axis.

Differences in habitat types were examined by tick species with sufficient surveillance events, including *H. longicornis* and *H. flava*. For *H. longicornis*, surveillance events were most frequently recorded in the surroundings of rural villages (33.7%) and country parks (18.3%), whereas for *H. flava*, events were primarily recorded in urban parks (24.8%) and forest parks (20.0%). Tick species significantly differed by habitat type (Pearson’s χ² = 47.934, *P* < 0.001; Fisher-Freeman-Halton exact test, *P* < 0.001). Detailed outputs were presented in S1 Table.

### Key environmental variables associated with tick distribution

After correlation screening, two independent MaxEnt models for the dominant tick species each incorporated seven environmental variables (S2 Table). Based on percent contribution, elevation (52.4%) and the maximum temperature of the warmest month (bio5, 33.9%) were the two most influential variables in the *H. longicornis* model. Precipitation of the driest month (bio14, 48.8%) and elevation (25.4%) contributed most strongly to the *H. flava* model. Response curves for the environmental variables identified in each model were generated to illustrate their relationships with predicted habitat suitability (Fig 3).

**Fig 3:**
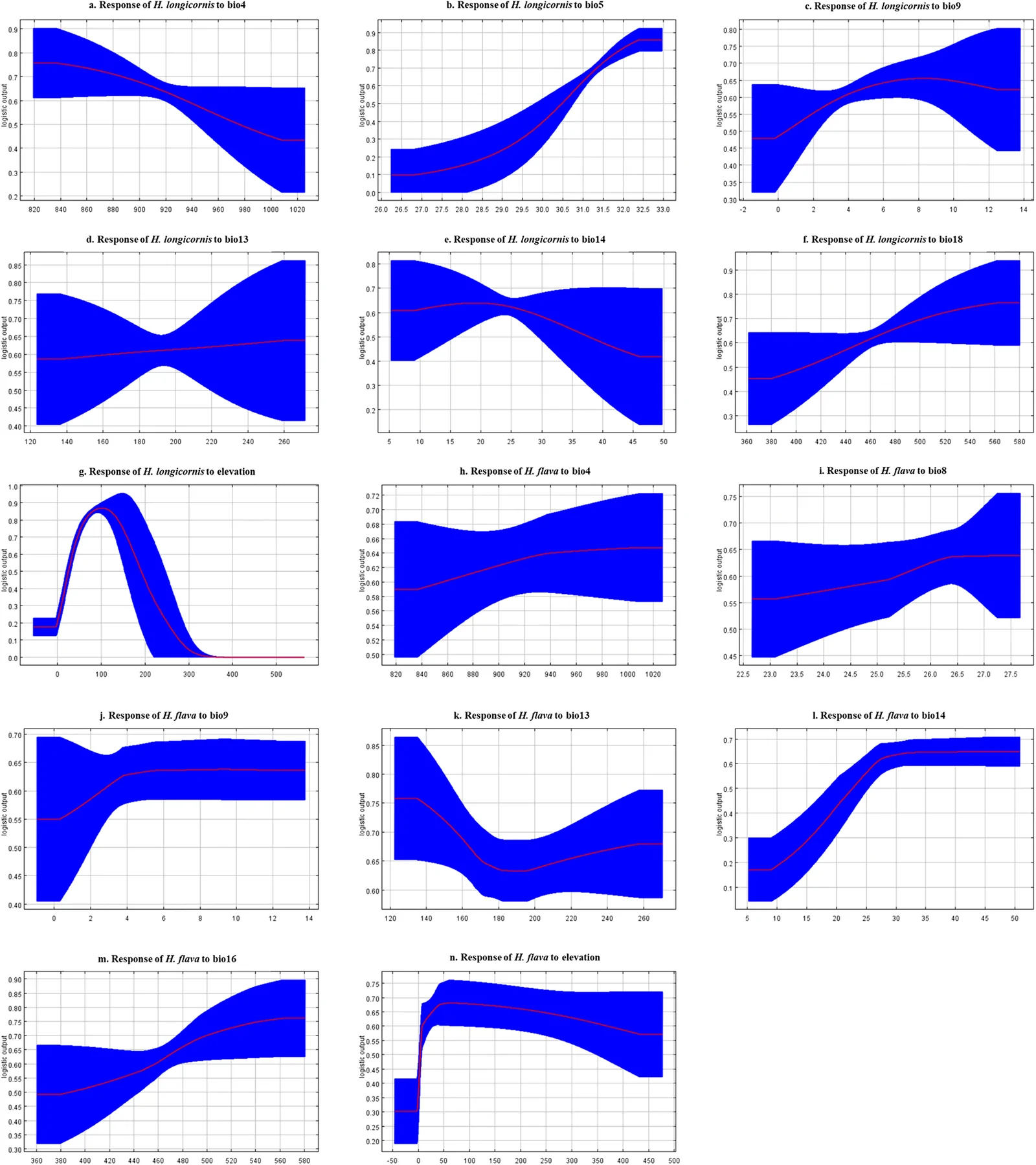
Response curves between predicted habitat suitability and the environmental variables. (a-g): *H. longicornis*; (h-n): *H. flava*. Biological meaning and contribution of environmental variables were provided in S2 Table.

For *H. longicornis*, predicted habitat suitability showed a unimodal response to elevation, increasing sharply at low elevations, peaking at intermediate elevations, and declining at higher elevations (Fig 3g). In contrast, suitability increased monotonically with bio5, with higher probabilities observed at higher temperatures (Fig 3b). For *H. flava*, predicted habitat suitability increased with bio14 and gradually approached a plateau at higher precipitation levels (Fig 3l). Suitability also varied with elevation, showing higher predicted probabilities at lower to intermediate elevations, followed by a slight decline at higher elevations (Fig 3n).

Jackknife analysis further showed that, for *H. longicornis*, elevation produced the highest model gain when used in isolation and resulted in the greatest decrease in gain when omitted from the full model. For *H. flava*, bio14 yielded the highest gain when used alone, whereas omitting elevation produced the largest reduction in model gain. Detailed results of variable contributions and Jackknife analysis were provided in S2 Table and S1 Fig.

### Predicted habitat suitability for dominant tick species

MaxEnt models were fitted for the two dominant tick species. For *H. longicornis*, the final model used RM = 1.5 with LQ as a feature-class combination, yielding an average AUC of 0.840 ± 0.070. For *H. flava*, the final model used RM = 2.0 and feature-class combination LQHP, with an average AUC of 0.818 ± 0.077. The optimized model with the lowest AICc (ΔAICc = 0) was selected for each species, compared to the default MaxEnt parameterization (S3 Table), for subsequent habitat suitability mapping and future projections.

Under current climatic conditions, areas classified as highly suitable for *H. longicornis* and *H. flava* were primarily concentrated in the southwestern hilly regions of Jiangsu Province (Fig 4a and 4d), indicating a geographically restricted core distribution.

**Fig 4:**
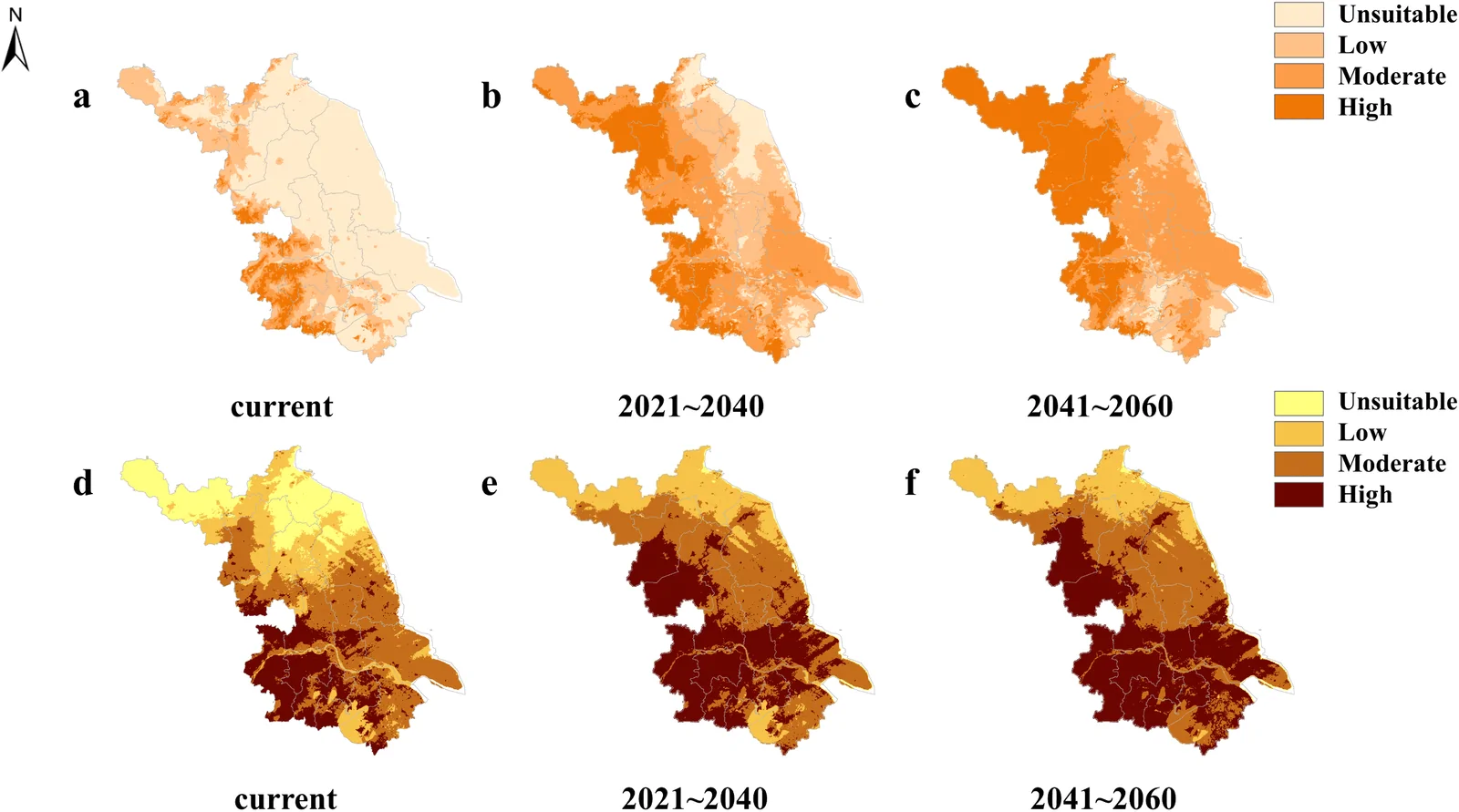
Predicted habitat suitability of two dominant tick species under current and future climate scenarios. (a-c): *H. longicornis*; (d-f): *H. flava.* Panels **a** and **d** represented current climatic conditions; panels **b** and **e** represented projections for 2021 ~ 2040; and panels **c** and **f** represented projections for 2041–2060. Habitat suitability was classified into four categories: unsuitable, low, moderate, and high, using Jenks natural breaks for each MaxEnt logistic output. High-suitability area (km²) was calculated for each scenario as the proportion of high-suitability pixels multiplied by the total area of Jiangsu Province (107,200 km²). The basic map is sourced from the Chinese Resource and Environmental Science Data Platform (http://www.resdc.cn/, https://doi.org/10.12078/2023010102).

Under the SSP245 climate scenario (SSP, Shared Socioeconomic Pathway), a larger area was classified as highly suitable for *H. longicornis* in both future periods under the scenario-specific Jenks classification. The area assigned to the high-suitability class increased from 5,465 km² under current conditions to 23,856 km² (a 336.5% increase) in 2021 ~ 2040, and further to 40,455 km² (a 640.3% increase) in 2041 ~ 2060 (Fig 4b and 4c). This pattern suggests spatial reorganization of predicted suitability from the southwestern highlands toward the eastern and northern parts of the province.

For *H. flava*, highly suitable habitats were already extensive under current climatic conditions (21,953 km²) and broadly distributed across the study area (Fig 4d). Future projections showed larger scenario-specific high-suitability class areas, reaching 42,015 km² (91.4% increase) in 2021 ~ 2040 and 48,037 km² (118.8% increase) in 2041 ~ 2060 (Fig 4e and Fig 4f), with increased spatial continuity across previously less suitable regions.

### Spatial concordance between publicly reported *SFTS* burden and projected tick suitability

To provide a disease-relevant ecological context for the vector distribution analysis, we summarized publicly reported *SFTS* burden across the 13 prefecture-level cities in Jiangsu Province and linked these epidemiological data with the predicted city-level tick habitat suitability in the present study (S4 Table). Published provincial surveillance data reported 914 *SFTS* cases in Jiangsu during 2014 ~ 2023, involving 49 counties. Nanjing and Huai’an were the two major endemic regions, with 489 and 236 reported cases, respectively, together accounting for 79.32% of all cases, whereas Nantong was the only prefecture-level city without reported *SFTS* cases. The remaining ten affected cities together accounted for 189 cases and were therefore classified as reported low-burden cities. The city-level comparison suggested a broad spatial concordance between reported *SFTS* burden and predicted habitat suitability for the two dominant tick species, particularly in major endemic areas such as Nanjing and Huai’an. In Nanjing, which had a high *SFTS* burden, both *H. longicornis* and *H. flava* showed high suitability indices. Similarly, southern Huai’an also exhibited high suitability indices for both dominant tick species. For several other cities with lower reported *SFTS* burdens, predicted tick suitability was generally moderate or low, although city-level concordance was not expected to be perfect because human *SFTS* occurrence also depends on virus circulation, host ecology, human exposure, and surveillance sensitivity.

## Discussion

Tick-borne diseases, including *SFTS*, continue to pose growing public health challenges in East Asia [37,38]. In this province-wide assessment of Jiangsu, we used six years of field surveillance to link the observed seasonal and habitat-specific patterns of dominant tick species with their current and future climatic suitability. This design allowed us to move beyond static occurrence mapping and to identify under which environmental conditions *H. longicornis* and *H. flava* are most likely to occur. Jiangsu represents an important setting for such an assessment because it is an *SFTS*-endemic province in eastern China, with a documented case-fatality rate of 7.44% [22], and is geographically connected to neighboring provinces such as Anhui, Shandong, and Zhejiang, where *SFTS* cases have also been reported [39–41]. By combining long-term local surveillance with climate-based distribution modeling, our findings provide regionally grounded evidence on tick population dynamics and potential future range shifts. This evidence may help refine surveillance priorities, guide seasonal and habitat-targeted tick control, and improve understanding of climate-sensitive *SFTS* risk across eastern China and the wider East Asian region.

In this study, we observed seasonal variation in monthly tick density for both *H. longicornis* and *H. flava*, with post-larval ticks concentrated in spring, particularly in April, and larvae peaking in autumn, particularly in September. In addition, owing to the activity of adult ticks [42], *H. longicornis* density remained relatively high during summer, especially in June and July, while *H. flava* also showed a third density peak during this period. This pattern, characterized by a spring peak of post-larval ticks and the highest larval density in September, was broadly consistent with the stage-specific seasonal dynamics reported in South Korea [43]. However, the positive correlation between tick density and surveillance events suggests that the observed seasonal distribution may partly reflect variation in sampling intensity across months. Even so, the provincial-level seasonal pattern identified in this study remains informative and provides a useful basis for future work aimed at further characterizing tick seasonal distribution.

Beyond seasonal variation, habitat types differed significantly (*P* < 0.001) between the two dominant tick species, indicating that tick surveillance events were not evenly distributed across landscape types, as previously reported in East Asia [44]. A previous study documented that the risk of *SFTS* transmission in Jiangsu Province is closely correlated with the spatial distribution and seasonal activity of local tick vectors [45]. Taken together, obvious seasonal patterns and distinct habitat types imply that tick population dynamics are regulated by both climatic seasonality and landscape structure at the provincial scale. These findings may provide essential data and evidence to inform tailored prevention and control measures for tick-borne diseases.

We identified potential environmental variables that affect tick distribution and found clear ecological relevance. This suggests that the combined influence of multiple environmental and climatic factors might shape the distributions of dominant tick species in the study area. Elevation contributed substantially to the distribution of both dominant tick species. Response curves indicated higher habitat suitability at low to intermediate elevations, a pattern comparable to findings reported in previous studies in Ningxia, China [46]. Bio5 represents thermal conditions during the warmest period of the year, which generally correspond to critical developmental stages in tick life cycles [47,48]. In the seasonal climatic context of the study area, this period coincides with summer, the peak season for blood feeding and mating in adult *H. longicornis*. Consequently, the increased temperature during this period may enhance tick activity and egg hatching efficiency. This is consistent with observations in Zhou et al.’s study [49], where warmer temperatures during the peak season have been shown to directly increase tick fitness and population expansion. Similarly, bio14 represents moisture conditions during the driest period, this period coincides with winter in the study area, when the tick is in a state of diapause or quiescence, sheltering in leaf litter to avoid freezing temperatures and desiccation, and relatively higher values of moisture may indicate environments that reduce desiccation stress and support overwintering persistence [50]. This pattern was consistent with findings from the studies by Yang et al. [32] and Zhao et al. [33]. In all, our findings suggest that temperature, moisture, and landscape structure jointly constrain tick habitat suitability at the provincial scale. Because tick populations cannot be fully eliminated, it warrants further study of how to optimize ecological measures to control tick populations and thereby contain tick-borne diseases.

Our predicted suitability patterns were broadly consistent with previous national-scale modeling studies, but also revealed province-specific details that are important for local surveillance. Yang et al. [32] projected that, under current climate and land-use conditions, suitable habitats for *H. longicornis* in Jiangsu were concentrated mainly in the northern and southwestern parts of the province, a pattern that is partly consistent with the high-suitability areas identified in our study. Ding et al. [34] similarly suggested that, under current and 2030s climate scenarios, most areas of Jiangsu had moderate to high *SFTS*-related risk, whereas the southeastern part of the province showed relatively lower risk. These findings support our observation that tick habitat suitability in Jiangsu is spatially heterogeneous rather than evenly distributed across the province. However, our future projections also indicated that highly suitable habitats would gradually widen and become more continuous, accompanied by a redistribution away from the southwestern hilly areas toward eastern and northern Jiangsu. This differs from Yang et al., who projected limited future change in *H. longicornis* suitability within Jiangsu, and from Ding et al., who projected a marked decline in *H. longicornis* suitability after the 2050s, with only localized hotspots remaining in north-eastern Jiangsu. These discrepancies may be related to differences in the underlying data, environmental variables, and modeling details. In particular, our model was calibrated using multi-year, province-wide field surveillance data on free-living ticks, with a specific focus on local habitat associations and ecological gradients within Jiangsu, whereas previous studies were generally conducted at broader spatial scales using compiled occurrence records or human case data. For *H. flava*, the slight mismatch between observed surveillance sites and predicted suitability may partly reflect uneven surveillance effort and the presence-only nature of MaxEnt, which estimates potential ecological suitability from environmental similarity rather than reproducing the observed sampling distribution. Although suitable areas appeared more concentrated in central and southwestern Jiangsu under current conditions, scattered suitability in eastern and northern Jiangsu became more evident under future scenarios.

The high-suitability areas identified for the two species in this study were concentrated mainly in the southwestern hilly region of Jiangsu, a pattern that is consistent with independently reported *SFTS* epidemiological patterns in the province. Tao et al. [22] analyzed notified *SFTS* cases from 2014 to 2023 and showed that cases were highly concentrated in Nanjing and Huai’an, with high-incidence counties largely located along the same southwestern hilly belt. This region includes low mountain and hilly landscapes, forest-grass ecotones, and rural environments that may provide suitable conditions for maintaining dominant tick populations. The spatial concordance between our predicted tick habitat suitability and reported *SFTS* epidemiology suggests that this broad ecological setting may be relevant to both vector persistence and the spatial concentration of *SFTS* cases.

However, this concordance should be interpreted as ecological context rather than direct evidence of *Bandavirus dabieense* circulation or human disease risk. Our study did not include *Bandavirus dabieense* detection in ticks, serological evidence from animal hosts or humans, or spatially matched analyses with county-level *SFTS* incidence. Human *SFTS* occurrence depends not only on vector habitat suitability, but also on local tick density, virus circulation in tick-host networks, animal host communities, human exposure behavior, and surveillance sensitivity. Nevertheless, from a One Health perspective, this vector ecological layer remains important because tick surveillance is a fundamental component of zoonotic disease prevention. By identifying where dominant tick species are seasonally abundant and environmentally suitable, our findings may help prioritize locations for active tick collection, *Bandavirus dabieense* testing, host surveillance, and risk characterization. Future studies should build on this ecological baseline by integrating tick infection testing, animal serology, and spatially resolved human case data to develop a more complete virus-vector-host-human framework for *SFTS* risk assessment and climate-sensitive early warning in eastern China, as such integrated evidence would be important for understanding *Bandavirus dabieense* circulation and its potential cross-regional spread.

The study had several limitations. First, tick surveillance data were temporally and spatially heterogeneous, with more surveillance events in April, May and September, and with relatively limited occurrence records in some areas. Therefore, the observed seasonal distribution may partly reflect sampling bias, and such heterogeneity may introduce uncertainty into model predictions. Second, to ensure the feasibility of future projections, only bioclimatic variables from the WorldClim database were included, thereby excluding potentially time-varying socio-economic factors (e.g., land use and host availability) and assuming these conditions remain unchanged in future scenarios. Third, the MaxEnt model estimates potential habitat suitability, which represent climate-driven habitat suitability rather than observed species presence or density.

## Methods

### Data source

This study was conducted in Jiangsu Province (Fig 5). Tick surveillance data were provided from Jiangsu Provincial Center for Disease Control and Prevention (CDC) between 2019 and 2024. The dataset comprised records of free-living ticks collected through routine provincial vector surveillance. Sentinel sites were selected by local CDCs based on surveillance feasibility and representation of major habitat types under the provincial vector surveillance framework. Each sentinel site was scheduled to be sampled at least once per season where feasible. Ticks were sampled using the standard drag/flag method [10], a widely applied technique for collecting questing ticks in the environment. Specimens were transported to the laboratory for species-level identification based on morphological characteristics by trained personnel. Representative original ventral micrographs of *H. longicornis* and *H. flava* nymphs and larvae are provided in S2 and S3 Fig. For each surveillance event, geographic coordinates, habitat type, and tick density (number of ticks per 100 meters of flagging distance) were systematically recorded. All data were organized and curated for subsequent statistical and ecological modeling analyses.

**Fig 5:**
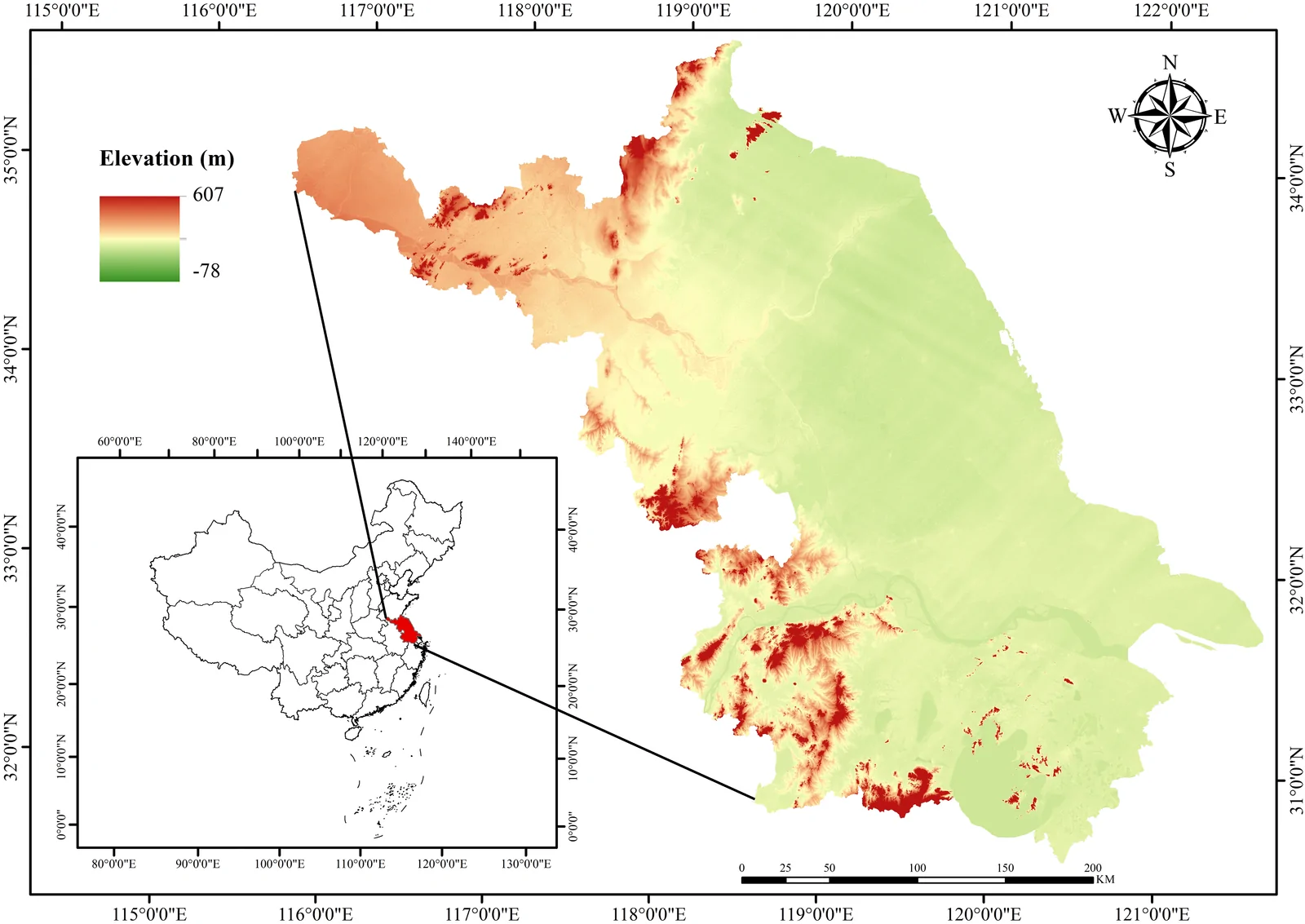
Geographical overview of Jiangsu Province. The basic map is sourced from the Chinese Resource and Environmental Science Data Platform (http://www.resdc.cn/, https://doi.org/10.12078/2023010102), and the elevation data is sourced from the SRTMDEM 90M-resolution DEM provided by Geospatial Data Cloud site, Computer Network Information Center, Chinese Academy of Sciences. (http://www.gscloud.cn).

### Tick surveillance analysis

Tick surveillance data were analyzed to characterize species diversity, seasonal dynamics, and habitat-associated patterns. Monthly seasonal dynamics were summarized as mean tick density (ticks per 100 m) for each calendar month. Larval and post-larval tick densities were summarized separately to describe life-stage-specific seasonal patterns, and the number of surveillance events was calculated as the number of sampling records in each month. Differences in monthly total tick density were assessed using one-way analysis of variance (ANOVA) after examination of distributional assumptions. Normality was evaluated using the Shapiro-Wilk test applied to ANOVA model residuals, and homogeneity of variances was assessed using a median-centered Levene’s test. When necessary, log transformation was applied to approximate normality and was used as a sensitivity analysis. Tukey’s honestly significant difference (HSD) test was specified for post-hoc multiple comparisons, and Welch’s ANOVA was calculated as a robustness check when applicable. To examine whether monthly surveillance intensity covaried with observed tick density, Pearson and Spearman correlation coefficients were calculated between the monthly number of surveillance events and monthly mean total tick density across all tick species.

Habitat types were classified into eight predefined categories: surroundings of rural villages, country parks, forest parks, grasslands on slopes, woodlands, urban parks, tidal flats, and urban residential areas. Species-specific habitat distribution was evaluated by comparing the frequency of surveillance events across habitat categories using the chi-square test (or Fisher’s exact test when appropriate). Statistical significance was defined as *P* < 0.05. All analyses were conducted using R version 4.5.2.

### Environmental variable selection and preprocessing

Environmental variables used for MaxEnt modeling were obtained from the WorldClim database [51] (https://www.worldclim.org/), including 19 bioclimatic variables (bio1-bio19) and elevation data, with a spatial resolution of 30 arc-seconds (approximately 1 km × 1 km). To evaluate climate-driven changes in tick habitat suitability, the same set of variables was derived from the BCC-CSM2-MR global climate model (CMIP6) under the SSP245 scenario for two future periods (2021 ~ 2040 and 2041 ~ 2060). All raster layers were clipped to the study area using ArcGIS 10.8, and resampled and aligned to ensure consistent spatial resolution and extent.

To minimize model overfitting due to multicollinearity, Pearson correlation analysis was conducted among the 19 bioclimatic variables and elevation. Correlation coefficients were calculated using environmental values extracted across the study area. Variables with an absolute correlation coefficient (|r|) ≥ 0.8 were considered highly correlated. For each correlated pair or cluster, one variable was retained based on ecological relevance and lower redundancy, while the others were excluded. The resulting subset of variables was used consistently for both current and future projections in the MaxEnt modeling.

### MaxEnt model construction and optimization

MaxEnt 3.4.4 was used to develop models for dominant tick species. Before model calibration, surveillance sites were processed in ArcGIS 10.8 using a buffer-based filtering procedure. A buffer radius of 10 km was applied to mitigate potential overfitting associated with spatial clustering, retaining a single surveillance site within each buffer [52]. Additionally, 10,000 background points were randomly generated across Jiangsu Province for model calibration.

Model tuning was conducted in R (version 4.5.2) using the ENMeval package (version 2.0.5) [36], implementing a block cross-validation approach to account for spatial structure in the occurrence data. Candidate models were generated by systematically testing combinations of regularization multiplier (RM) and feature classes (FC). RM values ranged from 0.5 to 4.0 (increment = 0.5), and four FC combinations were evaluated: L (linear), LQ (linear + quadratic), LQH (linear + quadratic + hinge), and LQHP (linear + quadratic + hinge + product). Model selection was primarily based on the corrected Akaike Information Criterion (ΔAICc), which balances model fit and complexity.

Using the optimal RM–FC combination, the final MaxEnt models were fitted with 10 bootstrap replicates. In each replicate, 25% of occurrence records were randomly withheld for internal model evaluation. Model performance was assessed using the area under the receiver operating characteristic curve (AUC). Model outputs were generated in logistic format and exported as raster layers for spatial analysis. Variable importance was evaluated using the jackknife test implemented in MaxEnt. Univariate response curves were examined to characterize relationships between environmental variables and predicted habitat suitability.

### Habitat suitability mapping and spatial extent analysis

MaxEnt logistic outputs (0 ~ 1) were reclassified into four suitability classes using Jenks natural breaks applied separately to each model output (current climate, 2021 ~ 2040, and 2041 ~ 2060). High-suitability area (km²) was calculated for each scenario as the proportion of high-suitability pixels multiplied by the total area of Jiangsu Province (107,200 km²). Because classification thresholds were derived independently for each projection, percentage changes in the high-suitability class reflect combined changes in predicted suitability distributions and scenario-specific class boundaries. These estimates should therefore be interpreted as changes in scenario-specific high-suitability class area, rather than expansion under a fixed suitability threshold.

## Supporting information

S1 Table Tick species and habitat types in Jiangsu Province, eastern China. (XLSX)

S2 Table Biological meaning and contribution of environmental variables. (XLSX)

S3 Table Parameters of the MaxEnt model with default and optimized settings. (XLSX)

S4 Table Spatial concordance between publicly reported *SFTS* burden and predicted tick suitability. (XLSX)

S1 Fig Regularized training gain for *Haemaphysalis longicornis* (A) and *Haemaphysalis flava* (B) by using the Jackknife test. (TIF)

S2 Fig Morphological Microscopic Features of *Haemaphysalis longicornis* and *Haemaphysalis flava* Nymphs. (TIF)

S3 Fig Morphological features of *Haemaphysalis longicornis* and *Haemaphysalis flava* larvae. (TIF)
