# Supplementary material for: Spatial and seasonal patterns of tick distribution and climate-driven shifts in Jiangsu Province, an SFTS-affected region of eastern China
Source: PLoS Negl Trop Dis. 2026 Nov 4;20(11):e0014847. doi: 10.1371/journal.pntd.0014847 (PMC13754229; doi:10.1371/journal.pntd.0014847)
Supplement: Supplementary file 1 [file pntd.0014847.s008.docx]

This study presents a well‑conducted analysis of long‑term surveillance data (2019–2024) on free‑living ticks in Jiangsu Province, eastern China. The authors characterize species composition, seasonal dynamics, habitat preferences, and use an optimized MaxEnt modeling framework to assess current and future climate‑driven changes in habitat suitability for the two dominant tick species, *Haemaphysalis longicornis* and *Haemaphysalis flava*. The work is timely, methodologically sound, and provides valuable insights for SFTS prevention within a One Health framework. The manuscript is generally clear, but several minor issues should be addressed before acceptance.

1. Language and grammar – Minor language polishing is recommended. Examples:

Abstract: “A total of 8,860 ticks belonging to seven species across three genera were identified” – fine, but later “2.854 ticks/100 m” should be “2.854 ticks/100 m²” or clarify the unit.

Methods (Section 2.5): “Jenks natural breaks method” – ensure consistent capitalization (“Jenks natural breaks” is acceptable).

Discussion: “That projected climate change may substantially reshape…” – the sentence structure is awkward; revise for clarity.2. Terminology consistency – The terms “transovarial transmission efficiency” and “transstadial transmission efficiency” are defined only once in Methods. Please briefly restate their definitions in the Results section for reader convenience.

2. Statistical details – In Section 3.1, one‑way ANOVA is used to compare monthly tick densities. Please report whether the assumptions of normality and homogeneity of variances were checked (e.g., using Shapiro‑Wilk or Levene’s test) and specify the post‑hoc test used if multiple comparisons were performed.

3. Habitat classification – Eight habitat types are listed (e.g., “surroundings of rural villages”, “country parks”, “forest parks”). Provide clear operational definitions or examples for each category to improve reproducibility.

4. Spatial bias handling – The authors mention spatial filtering to reduce sampling bias (Boria et al. 2014). Please specify the distance threshold used for spatial thinning of occurrence records.

5. MaxEnt tuning details – The regularization multiplier (RM) range is given as 0.5 to 4.0 (increment 0.5). Please also report the number of background points used and whether they were spatially constrained (e.g., within a buffer around occurrence points).

6. Model evaluation – AUC values are reported (0.840 for *H. longicornis*, 0.818 for *H. flava*). While acceptable, please also report the standard deviation or 95% confidence intervals from the 10 bootstrap replicates to indicate model robustness.

7. Spatial extent analysis – The increase in highly suitable areas for H. longicornis is reported as 336.5% (2021–2040) and 640.3% (2041–2060). Please verify these percentages against the absolute areas (e.g., current highly suitable area = ? km²). The numbers seem very high; check if the baseline area is very small initially.

8. Discussion of transmission threshold – The authors propose defining an “operational trigger threshold” based on tick density quartiles or deciles. This is an interesting suggestion but remains hypothetical. Please rephrase to avoid overinterpretation, or add a brief caveat that such thresholds require field validation.

9. Limitations – The limitations section acknowledges spatial heterogeneity and the exclusive use of bioclimatic variables. Please also acknowledge that the MaxEnt model predicts habitat suitability, not actual tick presence or abundance, and that future projections assume no changes in land use or host availability.

10. Typographical / formatting errors –

Page 12, line “1 1 1 1...” – appears to be a formatting artifact from the PDF conversion. Please clean up the final manuscript to remove these repeated numbers.

In the Abstract, “2.854 ticks/100 m” – use unspaced “/” consistently; also “336.5%” and “640.3%” – space before “%” is usually not used in English; check journal style.

Figure 1 legend: the source URL is included – ensure that the map permission statement (GS (2024) 0650) complies with PLOS NTDs requirements.
