# Supplementary material for: Spatial and seasonal patterns of tick distribution and climate-driven shifts in Jiangsu Province, an SFTS-affected region of eastern China
Source: PLoS Negl Trop Dis. 2026 Nov 4;20(11):e0014847. doi: 10.1371/journal.pntd.0014847 (PMC13754229; doi:10.1371/journal.pntd.0014847)
Supplement: Supplementary file 2 [file pntd.0014847.s010.docx]

**Response to Reviewers for PNTD-D-26-00682**

**Dear Editors and Reviewers,**

We would like to sincerely thank you for taking the time to review our manuscript and for providing thoughtful and constructive comments. We truly appreciate the opportunity to revise our manuscript for consideration in this journal. We found the Reviewers’ comments and suggestions enormously helpful in improving the quality and clarity of our work. We have carefully addressed all comments and revised the manuscript accordingly. Detailed point-by-point responses are provided below.

**Response to Reviewers**

**Reviewer #1**

**Methods**

*** The sampling strategy for sentinel sites and the survey frequency at each site should be clearly described.**

**Authors’ response:** Thank you for your valuable comment. We agree that a clearer description of the sentinel site sampling strategy and survey frequency would improve the understanding of the sampling design.

We have revised the **Methods - Data source** to provide additional details. We clarified that sentinel sites were selected by local CDCs based on surveillance feasibility and representation of major habitat types under the annual provincial vector surveillance framework and were designed to represent different habitat types. Each sentinel site was scheduled to be sampled for ticks at least once per season.

**The revised text now reads:**

“The dataset comprised records of free-living ticks collected through routine provincial vector surveillance. Sentinel sites were selected by local CDCs based on surveillance feasibility and representation of major habitat types under the provincial vector surveillance framework. Each sentinel site was scheduled to be sampled at least once per season where feasible”.

*** The method used to select absence (or pseudo-absence) locations for ENM should be specified.**

**Authors’ response:** Thank you for this important comment. We would like to clarify that, unlike many presence–absence ENM approaches that require pseudo-absence data, MaxEnt is a presence-only modeling framework and does not use absence or pseudo-absence locations. Instead, it relies on background points to characterize the environmental conditions available within the study area. In this study, 10,000 background points were randomly generated across Jiangsu Province and used as environmental reference points during model calibration. This clarification has been added to the **Methods - MaxEnt model construction and optimization**.

**The revised text now reads:**

“Additionally, 10,000 background points were randomly generated across Jiangsu Province for model calibration”.

*** As an optional suggestion: given that multiple tick species are analyzed, a joint species distribution model could provide additional insights into potential interspecies interactions.**

**Authors’ response:** We appreciate this suggestion. Because the present study focuses on the two dominant tick species and the remaining species had sparse occurrence records, a joint species distribution model would not be sufficiently supported by the current dataset. We therefore retained species-specific MaxEnt models and have added this as a direction for future work.

**Results**

*** The total number of sampling sites and the number of presence records for each species should be reported.**

**Authors’ response:** Thank you for pointing out this omission in the presentation of our results. We agree that the total number of sampling sites and the number of presence records for each species should be clearly reported. We have added them in the **Result**s - **Characterization of Tick Distribution** and species-specific presence records in **Table 1**.

And we would like to explain that for consistency in terminology throughout the manuscript, we refer to sampling sites as “surveillance sites” and presence records as “surveillance events” in the revised manuscript.

**The revised text and Table 1:**

“Between 2019 and 2024, a total of 7 tick species, belonging to 3 genera, were identified through routine surveillance in 256 surveillance sites across Jiangsu Province”.

**Table 1.** Tick species identified in Jiangsu Province, eastern China, in 2019-2024

| **Family** | **Genus** | **Species** | **Number** | **Surveillance events** |
| --- | --- | --- | --- | --- |
| **Ixodidae** | ***Haemaphysalis*** | *H. longicornis* | 7897 | 404 |
|  |  | *H. flava* | 868 | 123 |
|  |  | *H. phasiana* | 90 | 18 |
|  |  | *H. campanulata* | 1 | 2 |
|  |  | *H. hystricis* | 1 | 1 |
|  | ***Rhipicephalus*** | *R. microplus* | 2 | 1 |
|  | ***Ixodes*** | *I. sinensis* | 1 | 1 |

*** Figure 2: Consider using point size or a color gradient to represent tick density across locations, which may also help validate ENM outputs.**

**Authors’ response:** We thank the reviewer for this constructive suggestion. We agree that using point size or a color gradient to represent tick density can provide an intuitive visualization of spatial variation in observed tick abundance. We carefully considered this option during revision.

Because the present data were collected through routine sentinel surveillance rather than spatially complete probability-based sampling, surveillance effort was uneven across locations and habitat types. Under this condition, a density-coded map may be visually informative, but it could also be misinterpreted as a complete spatial representation of tick abundance across Jiangsu Province. To avoid overrepresenting the spatial completeness of the observed data, we retained a point-based visualization in the main figure, which more faithfully preserves the original surveillance structure and occurrence records used for MaxEnt modelling.

To improve clarity, we have revised the Figure 2 legend to explicitly state that the map shows surveillance sites with recorded tick occurrence, rather than interpolated or area-level tick density. We also clarified in the Methods and Discussion that surveillance data were spatially heterogeneous and that modeled habitat suitability should be interpreted as potential ecological suitability rather than observed tick abundance.

*** Figure 3: As the surveillance data are a key strength of this study, this figure could be expanded to include stratification by tick species and life stage. For example, H. longicornis typically shows an autumn peak corresponding to larvae, often with much higher densities than nymphs (spring) and adults (summer). The current pattern raises questions about data consistency or whether specific protocols were used for larval sampling.**

**Authors’ response:** We thank the reviewer for this valuable suggestion. We agree that the surveillance data represent an important strength of this study and that stratification by species and life stage can provide additional ecological insights.

The surveillance database used in this study recorded two developmental-stage categories: **larval** and **post-larval** ticks (nymphs and adults are combined).

Following the reviewer's suggestion, we stratified the monthly density data by species and developmental stage and added seasonal activity curves for both *H.longicornis* and *H.flava* (As **Table 1** showed, another species did not have adequate records).

Although the surveillance records did not allow us to further distinguish nymphs from adults, the observed seasonal patterns were broadly consistent with the expected tick life cycle. For both *H. longicornis* and *H. flava*, the autumn peak was primarily driven by larvae, whereas the spring peak was dominated by post-larval ticks. These findings support the biological consistency of the surveillance data and help explain the overall seasonal dynamics shown in **Figure 3**.

We have also expanded the **Results- Characterization of tick distribution** to describe the revised figure.

Thank you again and we really appreciate this insightful suggestion.

**The revised Figure 3:**

**Figure 3**. Seasonal dynamics of tick density and surveillance events in Jiangsu Province, 2019-2024. (a) *H. longicornis*; (b) *H. flava*. Monthly mean tick density (ticks per 100 m) is shown by lines, including total density (black), larval density (green), and post-larval density (red). Post-larval ticks comprise nymphs and adults. Light grey bars represent the number of surveillance events for each month and are plotted on the secondary y-axis.

*** Figure 4: Only two response curves are shown, despite including seven environmental variables. It would be more informative to present all response curves.**

**Authors’ response:** We thank the reviewer for this valuable suggestion. In response, we have added response curves for all environmental variables included in the model to **Figure 4** to improve completeness and interpretability of the results. The updated **Figure 4** now presents: (a-g) response curves of *H. longicornis* and (h-n) response curves of *H. flava*.

The revised **Figure 4**:

**Figure 4**. Response curves generated by the MaxEnt model illustrating the relationship between predicted habitat suitability and the environmental variables for each dominant tick species. (a-g): *H. longicornis*; (h-n): *H. flava.*

*** Figure 5: The classification criteria for “unsuitable,” “low,” “moderate,” and “high” suitability should be clarified. Additionally, panel (d) suggests that H. flava may be suitable in the southwest of Jiangsu, which appears inconsistent with Figure 1—this discrepancy should be explained.**

**Authors’ response:** We thank the reviewer for this helpful comment.

First, for each species, MaxEnt logistic outputs (0~1) were reclassified into four suitability classes using Jenks natural breaks applied separately to each model output (current climate, 2021~2040, and 2041~2060). High-suitability area (km²) was calculated for each scenario as the proportion of high-suitability pixels multiplied by the total area of Jiangsu Province (107,200 km²). We clarified this point in **Methods** and **Figure 5** caption for easier reference.

Second, we acknowledge the slight discrepancy between the observed surveillance sites in **Figure 1** and the predicted suitability for *H. flava* in **Figure 5d**. In **Figure 1**, *H. flava* surveillance records were mainly concentrated in the central part of Jiangsu Province, with some occurrences in the eastern region, whereas **Figure 5d** suggests relatively higher suitability in parts of southwestern Jiangsu. We clarified that this pattern should be interpreted in light of the presence-only nature of MaxEnt and the uneven distribution of surveillance effort. MaxEnt estimates potential ecological suitability based on environmental similarity to known occurrence records, rather than directly reproducing the observed spatial distribution of sampling sites. **Therefore, areas with few or no surveillance records may still be predicted as suitable if their environmental conditions resemble those associated with known occurrences.** We also note that, although the current suitability map shows more visually concentrated suitable areas in central and southwestern Jiangsu, **smaller suitable areas are also present in eastern and northern Jiangsu**, and these areas become more evident under future climate scenarios.

We have clarified the classification procedure in the **Methods** and added an explicit explanation of the observed–predicted discrepancy in the **Discussion**. We thank the reviewer again for this constructive comment.

**The revised text now reads:**

**Methods**

**Habitat suitability mapping and spatial extent analysis**

“MaxEnt logistic outputs (0~1) were reclassified into four suitability classes using Jenks natural breaks applied separately to each model output (current climate, 2021~2040, and 2041~2060). High-suitability area (km²) was calculated for each scenario as the proportion of high-suitability pixels multiplied by the total area of Jiangsu Province (107,200 km²). Because classification thresholds were derived independently for each projection, percentage changes in the high-suitability class reflect combined changes in predicted suitability distributions and scenario-specific class boundaries. These estimates should therefore be interpreted as changes in scenario-specific high-suitability class area, rather than expansion under a fixed suitability threshold.”

**Discussion**

**“**For *H. flava*, the slight mismatch between observed surveillance sites and predicted suitability may partly reflect uneven surveillance effort and the presence-only nature of MaxEnt, which estimates potential ecological suitability from environmental similarity rather than reproducing the observed sampling distribution. Although suitable areas appeared more concentrated in central and southwestern Jiangsu under current conditions, scattered suitability in eastern and northern Jiangsu became more evident under future scenarios”.

**Conclusions**

**Title**

*** The title refers to “spatiotemporal dynamics,” but the manuscript primarily reports seasonality and spatial distribution, rather than true spatiotemporal dynamics. The title may need to be revised for accuracy.**

**Authors’ response:** We thank the reviewer for this insightful comment. We agree that the original title may have overstated the temporal component by implying fully resolved spatiotemporal dynamics. In the revised manuscript, we have therefore modified the title to more accurately reflect the study’s scope, which focuses on spatial distribution patterns and seasonal variation, together with climate-driven changes in tick distribution.

**The revised title:**

Spatial and seasonal patterns of tick distribution and climate-driven shifts in an SFTS-affected region of eastern China

**Introduction**

*** Previous ENM studies of H. longicornis should be more comprehensively reviewed. The authors should clearly state the novelty of the current study relative to existing work and explicitly define the research gap being addressed. Relevant studies include, but are not limited to:**

* Yang X, Gao Z, Wang L, Xiao L, Dong N, Wu H, Li S. Projecting the potential distribution of ticks in China under climate and land use change. International Journal for Parasitology. 2021 Aug 1;51(9):749-59.;

* Zhao GP, Wang YX, Fan ZW, Ji Y, Liu MJ, Zhang WH, Li XL, Zhou SX, Li H, Liang S, Liu W. Mapping ticks and tick-borne pathogens in China. Nature communications. 2021 Feb 17;12(1):1075.;

* Zhao L, Li J, Cui X, Jia N, Wei J, Xia L, Wang H, Zhou Y, Wang Q, Liu X, Yin C. Distribution of Haemaphysalis longicornis and associated pathogens: analysis of pooled data from a China field survey and global published data. The Lancet Planetary Health. 2020 Aug 1;4(8):e320-9.;

* Ding FY, Ge HH, Ma T, Wang Q, Hao MM, Li H, Zhang XA, Maude RJ, Wang LP, Jiang D, Fang LQ. Projecting spatiotemporal dynamics of severe fever with thrombocytopenia syndrome in the mainland of China. Global Change Biology. 2023 Dec;29(23):6647-60.

**Authors’ response:** Thank you for this important suggestion. We have revised the Introduction to provide a more comprehensive review of previous ecological niche modeling studies relevant to H. longicornis and tick distribution in China. In the revised text, we now summarize national-scale MaxEnt-based studies that projected the potential distributions of multiple tick species, studies linking projected H. longicornis suitability with SFTS incidence under climate-change scenarios, and recent county-level mapping work integrating tick species, tick-borne agents, and human infection records.

We also explicitly define the remaining research gap: most existing maps and models have been developed at broad spatial scales and often rely on compiled occurrence records, pathogen detections, or reported human cases, which may not fully capture the seasonal dynamics, habitat-specific patterns, and local ecological heterogeneity of free-living ticks within a single SFTS-affected province. We further clarified that the novelty of the present study lies in integrating multi-year, locally collected surveillance data with province-scale modeling in Jiangsu Province, rather than in applying ecological niche modeling alone.

**The revised Introduction:**

“Ecological niche models (ENMs), especially the maximum entropy model (MaxEnt), are widely used to project species distributions under current and future climate scenarios [29-31]. In China, Yang et al. [32] and Zhao et al. [33] used tick occurrence records from public datasets and published studies with the MaxEnt model to project the potential distributions of multiple tick species at the national scale. Ding et al. [34] further linked projected H.longicornis suitability with SFTS case data in a multi-model, multi-scenario framework, revealing spatially heterogeneous changes in vector suitability and SFTS incidence under climate change. In addition, the county-level tick and pathogen atlas developed by Zhao et al. [35] integrated records for 124 tick species, 103 tick-borne agents, and human infections associated with 29 pathogen species or subspecies, and showed that predicted suitable habitats for predominant vector ticks were substantially broader than documented occurrence areas. Together, these studies have provided important national-scale evidence on tick diversity, tick-borne pathogens, and climate-sensitive distribution risks, with clear implications for public health planning and policy. However, most existing maps and models have been developed at relatively broad spatial scales, often relying on compiled occurrence records, pathogen detections, or reported human cases. As a result, they may not fully capture the seasonal dynamics, habitat-specific patterns, and local ecological heterogeneity of free-living ticks within a single SFTS-affected province. Their direct transferability to tick surveillance and SFTS prevention in Jiangsu Province therefore remains limited.

To address this gap, we analyzed province-wide surveillance data on free-living ticks collected in Jiangsu Province from 2019 to 2024. We characterized the seasonal and habitat-specific distribution patterns of dominant tick species and estimated their potential spatial distributions under current and future climatic conditions using an optimized MaxEnt framework [36]. The novelty of this study lies not in the application of ecological niche modeling alone, but in the integration of multi-year, locally collected surveillance data with province-scale modeling in an SFTS-affected region of eastern China. By linking field surveillance evidence with climate projections for H. longicornis and H. flava, this study provides a locally calibrated and spatially explicit assessment of tick habitat suitability of the two dominant species that may inform climate-sensitive surveillance, targeted vector control, and SFTS prevention strategies in Jiangsu and comparable eastern Chinese settings”.

**Discussion**

*** In paragraph 3, in addition to the two peaks discussed, there is typically a summer peak corresponding to adult activity, which should be acknowledged.**

**Authors’ response:** We thank the reviewer for this helpful comment. We agree that, in addition to the spring and autumn peaks, the summer increase associated with adult tick activity should be acknowledged. We have revised the **Discussion** to explicitly mention that *H. longicornis* density remained relatively high during summer and that *H. flava* also showed an additional summer peak.

**The revised text in discussion now reads:**

“In this study, we observed pronounced seasonal variation in monthly tick density for both *H. longicornis* and *H. flava*, with post-larval ticks concentrated in spring, particularly in April, and larvae peaking in autumn, particularly in September. In addition, owing to the activity of adult ticks [36], *H. longicornis* density remained relatively high during summer, especially in June and July, while *H. flava* also showed a third density peak during this period. This pattern, characterized by a spring peak of post-larval ticks and the highest larval density in September, was broadly consistent with the stage-specific seasonal dynamics reported in south Korea [37]”.

*** The predicted suitability patterns and response curves should be compared more explicitly with findings from previous studies, highlighting consistencies or discrepancies.**

**Authors’ response:** We thank the reviewer for this helpful comment. We agree that the predicted suitability patterns and response curves should be interpreted more explicitly in relation to previous studies. In the revised Discussion, we have added a comparison with relevant national-scale modeling studies, particularly those by Yang et al. and Ding et al. We now describe where our predicted suitability patterns are consistent with previous findings, including the concentration of suitable habitats for *H. longicornis* in northern and southwestern Jiangsu and the relatively lower risk predicted for southeastern Jiangsu. We also highlight differences in future projections, noting that our results suggest gradual expansion and increased spatial continuity of highly suitable habitats, whereas previous studies projected either limited future change or a decline in suitability after the 2050s.

In addition, we have expanded the discussion of response for key environmental variables. We compared the elevation response with previous findings from Ningxia, China, and added the discussion of how the responses to Bio5 and Bio14 may reflect biologically relevant thermal and moisture conditions during critical periods of the tick life cycle. Specifically, we explain that warmer conditions during the warmest period of the year may enhance adult activity, blood feeding, mating, and egg hatching, whereas higher precipitation during the driest period may reduce desiccation stress and support overwintering persistence.

These revisions now more clearly place our suitability predictions and environmental response patterns within the context of previous studies, while also explaining potential consistencies and discrepancies.

**The revised text in discussion now reads:**

“Response curves indicated higher habitat suitability at low to intermediate elevations, a pattern comparable to findings reported in previous studies in Ningxia, China [46]. Bio5 represents thermal conditions during the warmest period of the year, which generally correspond to critical developmental stages in tick life cycles [47, 48]. In the seasonal climatic context of the study area, this period coincides with summer, the peak season for blood feeding and mating in adult H. longicornis. Consequently, the increased temperature during this period may enhance tick activity and egg hatching efficiency. This is consistent with observations in Zhou et al.’s study [49], where warmer temperatures during the peak season have been shown to directly increase tick fitness and population expansion. Similarly, bio14 represents moisture conditions during the driest period, this period coincides with winter in the study area, when the tick is in a state of diapause or quiescence, sheltering in leaf litter to avoid freezing temperatures and desiccation, and relatively higher values of moisture may indicate environments that reduce desiccation stress and support overwintering persistence [50]. Such mode was consistent with findings from the studies by Yang et al. [32] and Zhao et al. [33]”.

“Our predicted suitability patterns were broadly consistent with previous national-scale modeling studies, but also revealed province-specific details that are important for local surveillance. Yang et al. [32] projected that, under current climate and land-use conditions, suitable habitats for H. longicornis in Jiangsu were concentrated mainly in the northern and southwestern parts of the province, a pattern that is partly consistent with the high-suitability areas identified in our study. Ding et al. [34] similarly suggested that, under current and 2030s climate scenarios, most areas of Jiangsu had moderate to high SFTS-related risk, whereas the southeastern part of the province showed relatively lower risk. These findings support our observation that tick habitat suitability in Jiangsu is spatially heterogeneous rather than evenly distributed across the province. However, our future projections also indicated a gradual expansion and increased spatial continuity of highly suitable habitats, with a shift from the southwestern hilly areas toward eastern and northern Jiangsu. This differs from Yang et al., who projected limited future change in H. longicornis suitability within Jiangsu, and from Ding et al., who projected a marked decline in H. longicornis suitability after the 2050s, with only localized hotspots remaining in north-eastern Jiangsu. These discrepancies may be related to differences in the underlying data, environmental variables, and modeling details. In particular, our model was calibrated using multi-year, province-wide field surveillance data on free-living ticks, with a specific focus on local habitat associations and ecological gradients within Jiangsu, whereas previous studies were generally conducted at broader spatial scales using compiled occurrence records or human case data. For H. flava, the slight mismatch between observed surveillance sites and predicted suitability may partly reflect uneven surveillance effort and the presence-only nature of MaxEnt, which estimates potential ecological suitability from environmental similarity rather than reproducing the observed sampling distribution. Although suitable areas appeared more concentrated in central and southwestern Jiangsu under current conditions, scattered suitability in eastern and northern Jiangsu became more evident under future scenarios”.

**Minor Comments**

*** Bandavirus dabieense (formerly Dabie bandavirus) should be italicized in the Introduction, and the updated nomenclature should be used consistently.**

**Authors’ response:** We thank the reviewer for pointing out the nomenclature issue. We have carefully revised the manuscript to ensure consistency with the updated taxonomy. The virus name has been updated to *Bandavirus dabieense*, and all occurrences throughout the manuscript have been checked and standardized.

**Summary and General Comments**

**This manuscript presents tick surveillance data from Jiangsu Province, one of the known hotspots for SFTS transmission in China, and models the potential distribution of H. longicornis and H. flava under future climate change scenarios. The tick surveillance dataset is novel and valuable, and could be further emphasized. While the ecological niche modeling (ENM) is also relevant, similar suitability mapping for these species has been conducted by multiple studies; therefore, the novelty of the methods and/or findings in the current study should be more clearly articulated.**

**Authors’ response:** Thank you for this important comment. We agree that the novelty of the manuscript should be framed more clearly around this dataset and its integration with ENM analysis. Accordingly, we have revised the Introduction to emphasize that Jiangsu Province is an SFTS-affected area in eastern China with sustained local transmission, and that our study uses province-wide surveillance data on free-living ticks collected from 2019 to 2024. We now state more explicitly that, although suitability mapping for *H. longicornis* and related tick species has been conducted previously in national scales, the contribution of this study is the combination of multi-year field surveillance, seasonal and habitat-specific characterization, and locally calibrated spatial modeling for the two dominant tick species, *H. longicornis* and *H. flava*.

**The revised Introduction:**

“Ecological niche models (ENM), especially the maximum entropy model (MaxEnt) is one of the most widely used techniques in projecting the spatial distribution of species under future climate scenarios [29-31]. In China, Yang et al. [32] and Zhao et al. [33] used tick occurrence records from public datasets and published studies with the MaxEnt model to project the potential distributions of multiple tick species at the national scale. Ding et al. [34] further linked projected H.longicornis suitability with SFTS case data in a multi-model, multi-scenario framework, revealing spatially heterogeneous changes in vector suitability and SFTS incidence under climate change. And the county-level tick and pathogen atlas developed by Zhao et al. [35] integrated records for 124 tick species, 103 tick-borne agents, and human infections associated with 29 pathogen species or subspecies, and showed that predicted suitable habitats for predominant vector ticks were substantially broader than documented occurrence areas. Together, these studies have provided important national-scale evidence on tick diversity, tick-borne pathogens, and climate-sensitive distribution risks, with clear implications for public health planning and policy. However, most existing maps and models have been developed at relatively broad spatial scales, often relying on compiled occurrence records, pathogen detections, or reported human cases. As a result, they may not fully capture the seasonal dynamics, habitat-specific patterns, and local ecological heterogeneity of free-living ticks within a single SFTS-affected province. Their direct transferability to tick surveillance and SFTS prevention in Jiangsu Province therefore remains limited.

To address this gap, we analyzed province-wide surveillance data on free-living ticks collected in Jiangsu Province from 2019 to 2024. We characterized the seasonal and habitat-specific distribution patterns of dominant tick species and estimated their potential spatial distributions under current and future climatic conditions using an optimized MaxEnt framework [36]. The novelty of this study lies not in the application of ecological niche modeling alone, but in the integration of multi-year, locally collected surveillance data with province-scale modeling in an SFTS-affected region of eastern China. By linking field surveillance evidence with climate projections for H. longicornis and H. flava, this study provides a locally calibrated and spatially explicit assessment of tick habitat suitability of the two dominant species that may inform climate-sensitive surveillance, targeted vector control, and SFTS prevention strategies in Jiangsu and comparable eastern Chinese settings”.

**Reviewer #2:**

**Methods**

**The objectives of the study had been clearly articulated with a clear testable hypothesis stated.**

**The study design needs to be updated to address the stated objectives.**

**The population has been clearly described and appropriate for the hypothesis being tested.**

**The sample size is sufficient to ensure adequate power to address the hypothesis being tested, additional data on the parasitic ticks would be appreciated.**

**The correct statistical analysis had been applied to to support conclusions.**

**The concerns about ethical or regulatory requirements have been met currently.**

**Authors’ response:** Thank you for your positive evaluation of our work. Regarding the update of the study design, we have carefully revised the **Introduction** section in response to all reviewers’ comments, with a clearer and more detailed articulation of the study objectives and its significance.

With respect to the parasitic tick data, we regret that we are currently unable to obtain and incorporate additional data at this stage. We sincerely appreciate your time and thoughtful assessment of our manuscript.

**Results**

**The analysis presented basically match the analysis plan. The results ae clearly and completely presented. The figures (Tables, Images) are presented with sufficient quality for clarity.**

**Authors’ response:** Thank you for your positive evaluation of our work.

**Conclusions**

**The conclusions are supported by the data presented. The limitations of analysis needs to be updated to clearly described. These data had been discussed to advance the understanding of the topic under current study. The public health relevance had been addressed in the manuscript.**

**Authors’ response:** Thank you for your positive evaluation of our work. We have revised the limitations section to clarify that future projections assume static land use and host availability, which may introduce additional uncertainty into long-term predictions, and the MaxEnt model estimates climatic habitat suitability rather than observed tick presence or abundance.

**The revised text now reads:**

“The study had several limitations. First, tick surveillance data were spatially heterogeneous, with relatively limited occurrence records in some areas, which may introduce uncertainty into model predictions. Second, to ensure the feasibility of future projections, only bioclimatic variables from the WorldClim database were included, thereby excluding potentially time-varying socio-economic factors (e.g., land use and host availability) and assuming these conditions remain unchanged in future scenarios. Third, the MaxEnt model estimates potential habitat suitability results, which represent climate-driven habitat suitability rather than observed species presence or density”.

**Summary and General Comments**

**In the manuscript, a provincial scale 5 years tick surveillance were performed in Jiangsu, Southeast China. Seasonal dynamics of tick communities and habitats-associated differences were characterized using an optimized MaxEnt modeling to reveal the determining environmental factors and hope to predict the potential health risk caused by ticks and tick-borne diseases. However, the current manuscript needs major revision before be accepted for publication.**

**1. Since the records of ticks and tick-borne diseases, especially for SFTS, in Jiangsu is paramount important to sponsor the current research, a brief and concise introduction of tick borne diseases in Jiangsu would be helpful.**

**Authors’ response:** We thank the reviewer for this helpful suggestion. We agree that a concise description of tick-borne diseases in Jiangsu Province, particularly SFTS, is important to better justify the rationale for the present study. Accordingly, we have revised the Introduction to summarize the current SFTS burden in Jiangsu Province. We also added recent local evidence on free-living tick species composition and tick-borne pathogen detection. These additions show that *H. longicornis* is consistently the dominant species while *H. flava* represents an important secondary species. We believe these revisions provide a clearer epidemiological and ecological background for the current province-wide tick surveillance and habitat suitability modeling study.

**The revised text in Introduction now reads:**

“Jiangsu Province, located along the lower reaches of the Yangtze River, is a recognized SFTS-affected area in eastern China and has reported sustained local transmission [22]. Between 2014 and 2023, a total of 914 SFTS cases were reported in Jiangsu Province, with an average annual incidence of 0.11 per 100,000 population and an overall case fatality rate of 7.44% [23]. Local tick and pathogen investigations in the region also point to persistent ecological risk. In Nanjing City, the capital of Jiangsu Province, a 2024 investigation found that free-living ticks were mainly *Haemaphysalis longicornis* (hereafter referred to as *H. longicornis*), accounting for 75.65%; and *Haemaphysalis flava* (hereafter referred to as *H. flava*) accounting for 23.91%. Pathogen testing showed a 25.49% positivity rate for Rickettsia in tick samples, and the *Bandavirus dabieense* seropositivity rate among animal hosts was 0.65% [24]. More recent city-level surveillance further showed a consistent dominance of *H. longicornis* and *H. flava* across Jiangsu Province: In Suzhou City, a study collected 148 ticks and found that *H. longicornis* (47.3%) was the predominant species, with spotted fever group Rickettsiae detected at a minimum infection rate of 2.8% [25]. Monitoring of free-living ticks in Yangzhou City from 2020 to 2024 found *H. longicornis* (70.01%) and *H. flava* (29.99%) as the only species detected [26], while surveillance in Xuzhou City from 2020 to 2023 reported *H. longicornis* as the dominant free-living tick species (92.08%) [27]. Together, these findings suggest that *H. longicornis* is consistently the dominant free-living tick species in Jiangsu, *H. flava* is an important secondary species”.

**2. The current surveillance had been performed targeting the off-host ticks, however, the parasitic ticks on domestical or wild animals would be highly recommended to be enrolled in the revised version.**

**Authors’ response:** We sincerely thank the reviewer for this valuable suggestion. We agree that data from parasitic ticks on domestic and wild animals would substantially strengthen the surveillance and provide a more complete picture of tick ecology and pathogen risk. However, our current surveillance was originally designed to focus on free-living ticks (off-host ticks), and we are unable to obtain additional parasitic tick samples or reliable historical data at this revision stage. Thank you again for your thoughtful suggestion.

**3. As H. flava was described as one of the main targets, the references of the species on the transmission of SFTSV would be cited.**

Jang et al., 2024 Microbiol Spectr 12: e00489-24.

Fang et al., 2023, Ticks and Tick-Borne Diseases 14, 102100

**Authors’ response:** We sincerely thank the reviewer for the recommendation. After carefully reading the references, we believe they are of great significance to support our study, and we have quoted them in the revised **Introduction** and **Discussion**.

Fang et al., 2023 in **Introduction**:

“Previous experimental studies have demonstrated that both H. longicornis and H. flava can acquire *Bandavirus dabieense* during blood feeding and subsequently support viral maintenance and transmission [28, **29**]”.

Jang et al., 2024 in **Discussion**:

“owing to the activity of adult ticks **[36]**, *H. longicornis* density remained relatively high during summer”.

**4. The Dabie Bandavirus had been proposed as “Bandavirus dabieense”,**

**Authors’ response:** We thank the reviewer for pointing out the nomenclature issue. We have carefully revised the manuscript to ensure consistency with the updated taxonomy. The virus name has been updated to *Bandavirus dabieense*, and all occurrences throughout the manuscript have been checked and standardized.

**Reviewer#3**

**This study presents a well-conducted analysis of long-term surveillance data (2019–2024) on free-living ticks in Jiangsu Province, eastern China. The authors characterize species composition, seasonal dynamics, habitat preferences, and use an optimized MaxEnt modeling framework to assess current and future climate-driven changes in habitat suitability for the two dominant tick species, Haemaphysalis longicornis and Haemaphysalis flava. The work is timely, methodologically sound, and provides valuable insights for SFTS prevention within a One Health framework. The manuscript is generally clear, but several minor issues should be addressed before acceptance.**

**1. Language and grammar – Minor language polishing is recommended. Examples:**

**Abstract:**

**“A total of 8,860 ticks belonging to seven species across three genera were identified” – fine, but later “2.854 ticks/100 m” should be “2.854 ticks/100 m²” or clarify the unit.**

**Authors’ response:** We thank the reviewer for this helpful suggestion. We have carefully revised the manuscript to ensure consistency and clarity in unit expression. The unit has been clarified as “ticks per 100 meters of flagging distance” to accurately reflect the drag sampling design, and this notation has been standardized：

“For each surveillance event, geographic coordinates, habitat type, and tick density (number of ticks per 100 meters of flagging distance) were systematically recorded.”

**Methods:**

**“Jenks natural breaks method” – ensure consistent capitalization (“Jenks natural breaks” is acceptable).**

**Authors’ response:** We thank the reviewer for this helpful suggestion. We have carefully revised the manuscript for consistency in terminology. In particular, we will uniformly use “**Jenks natural breaks**” throughout the manuscript to ensure clarity and consistency.

**The revised text now reads:**

“Predicted habitat suitability (ranging from 0 to 1) were classified into four categories (unsuitable, low, moderate, and high), using Jenks natural breaks to facilitate visualization and spatial comparison.”

**Discussion:**

**1.“That projected climate change may substantially reshape…” – the sentence structure is awkward; revise for clarity.**

**Authors’ response:** We thank the reviewer for this helpful comment. We have rewritten the **Discussion** and integrated the original sentence into a new paragraph. We will also examine the grammar structure of the entire text to avoid incorrect structures. Thank you again for your comment.

**Terminology consistency:**

**The terms “transovarial transmission efficiency” and “transstadial transmission efficiency” are defined only once in Methods. Please briefly restate their definitions in the Results section for reader convenience.**

**Authors’ response:** We thank the reviewer for this comment. However, we would like to clarify that the terms “transovarial transmission efficiency” and “transstadial transmission efficiency” are not used in our manuscript. We carefully checked the manuscript and confirmed that these terms do not appear in the current version. We have therefore not added definitions for these terms, but have further reviewed the manuscript to ensure consistent and clear terminology throughout.

**2. Statistical details:**

**In Section 3.1, one‑way ANOVA is used to compare monthly tick densities. Please report whether the assumptions of normality and homogeneity of variances were checked (e.g., using Shapiro‑Wilk or Levene’s test) and specify the post‑hoc test used if multiple comparisons were performed.**

**Authors’ response:** We thank the reviewer for this helpful comment. We have revised the **Methods - Tick surveillance analysis** to clarify how the assumptions for one-way ANOVA were assessed and to specify the post-hoc procedure used for multiple comparisons. Specifically, normality was evaluated using the Shapiro-Wilk test applied to ANOVA model residuals, and homogeneity of variances was assessed using a median-centered Levene's test. Tukey's honestly significant difference (HSD) test was specified for post-hoc multiple comparisons. We also clarified that log-transformed analyses were conducted as sensitivity analyses when raw-density residuals deviated from normality. Also, the **Results - Characterization of tick distribution** was updated.

**The revised text now reads:**

“Differences in monthly total tick density were assessed using one-way analysis of variance (ANOVA) after examination of distributional assumptions. Normality was evaluated using the Shapiro-Wilk test applied to ANOVA model residuals, and homogeneity of variances was assessed using a median-centered Levene’s test. When necessary, log transformation was applied to approximate normality and was used as a sensitivity analysis. Tukey’s honestly significant difference (HSD) test was specified for post-hoc multiple comparisons, and Welch's ANOVA was calculated as a robustness check when applicable.”

**3. Habitat classification:**

**Eight habitat types are listed (e.g., “surroundings of rural villages”, “country parks”, “forest parks”). Provide clear operational definitions or examples for each category to improve reproducibility.**

**Authors’ response:** We thank the reviewer for this helpful suggestion. We have now provided brief operational definitions for each habitat type and included them in Supplementary Materials (Table S1).

**The revised Table S1:**

**Table S1.** Tick species and habitat types in Jiangsu Province, eastern China

| **Habitat types** | **Definition** | ***Haemaphysalis longicornis*** | ***Haemaphysalis flava*** |
| --- | --- | --- | --- |
| Rural villages | Rural villages refer to low-density rural settlements consisting of scattered houses and small community infrastructures, typically surrounded by cropland, woodland, or mixed agricultural landscapes. | 136 | 23 |
| Country parks | Country parks are semi-natural recreational areas located at the urban fringe, characterized by high vegetation cover and managed natural landscapes, often including walking trails and basic visitor facilities. | 74 | 22 |
| Grasslands on slopes | Grasslands on slopes refer to natural or semi-natural grass-dominated habitats located on sloping terrain, usually with moderate to steep gradients, including degraded hillsides and open upland grass areas. | 56 | 9 |
| Forest parks | Forest parks are designated protected or managed forest areas with high canopy cover, primarily used for conservation and recreation, often containing natural or planted forests with limited human disturbance. | 56 | 25 |
| Woodlands | Woodlands refer to non-protected forested or semi-forested areas with tree cover, including fragmented secondary forests, shelterbelts, and small patches of natural or planted trees outside formal protected areas. | 42 | 10 |
| Urban parks | Urban parks are managed green spaces within urban or peri-urban areas designed for public recreation, typically consisting of landscaped vegetation, lawns, and constructed facilities. | 23 | 31 |
| Urban residential areas | Urban residential areas are high-density built-up environments dominated by residential buildings, roads, and other impervious surfaces, with limited natural vegetation cover. | 7 | 2 |
| Tidal flats | Tidal flats are intertidal coastal zones that are periodically exposed and submerged by tides, characterized by bare sediment surfaces or sparse salt-tolerant vegetation. | 10 | 3 |

**4. Spatial bias handling:**

**The authors mention spatial filtering to reduce sampling bias (Boria et al. 2014). Please specify the distance threshold used for spatial thinning of occurrence records.**

**Authors’ response:** We thank the reviewer for this comment. A 10 km buffer was applied for spatial thinning, within which only one occurrence was retained. This distance was used to reduce spatial clustering in surveillance data and to limit spatial autocorrelation while maintaining sufficient sample representation at the provincial scale. The method has now been clarified in the revised manuscript.

**The revised text now reads:**

“A buffer radius of 10 km was applied to mitigate potential overfitting associated with spatial clustering, retaining a single surveillance site within each buffer.”

**5. MaxEnt tuning details:**

**The regularization multiplier (RM) range is given as 0.5 to 4.0 (increment 0.5). Please also report the number of background points used and whether they were spatially constrained (e.g., within a buffer around occurrence points).**

**Authors’ response:** Thank you for this important comment. In this study, 10,000 background points were randomly generated across Jiangsu Province and used as environmental reference points during model calibration. This clarification has been added to the **Methods - MaxEnt model construction and optimization**.

**The revised text now reads:**

“Additionally, 10,000 background points were randomly generated across Jiangsu Province for model calibration.”

**6. Model evaluation:**

**AUC values are reported (0.840 for H. longicornis, 0.818 for H. flava). While acceptable, please also report the standard deviation or 95% confidence intervals from the 10 bootstrap replicates to indicate model robustness.**

**Authors’ response:** We agree with the reviewer that reporting model uncertainty is essential. Model performance was summarized as mean AUC ± SD across 10 bootstrap replicates after revision. The statistics are as follows:

H. longicornis: AUC = 0.840 ± 0.070; H. flava: AUC = 0.818 ± 0.077.

**The revised text now reads:**

For *H. longicornis*, the final model used RM = 1.5 with LQ as a feature-class combination, yielding an average AUC of 0.840 ± 0.070. For *H. flava*, the final model used RM = 2.0 and feature-class combination LQHP, with an average AUC of 0.818 ± 0.077.

**7. Spatial extent analysis:**

**The increase in highly suitable areas for H. longicornis is reported as 336.5% (2021–2040) and 640.3% (2041–2060). Please verify these percentages against the absolute areas (e.g., current highly suitable area = ? km²). The numbers seem very high; check if the baseline area is very small initially.**

**Authors’ response:** Thank you for your helpful comment. The baseline area is indeed relatively small, which partly explains the large percentage changes. This pattern is also reflected in the expansion of the dark orange highly suitable areas in Figure 5a-c.

**The revised text:**

Under the SSP245 climate scenario (SSP, Shared Socioeconomic Pathway), a larger area was classified as highly suitable for H. longicornis in both future periods under the scenario-specific Jenks classification. The area assigned to the high-suitability class increased from 5,465 km² under current conditions to 23,856 km² (a 336.5% increase) in 2021~2040, and further to 40,455 km² (a 640.3% increase) in 2041~2060 (Figures 5b and Figures 5c). This pattern suggests spatial reorganization of predicted suitability from the southwestern highlands toward the eastern and northern parts of the province.

**8. Discussion of transmission threshold:**

**The authors propose defining an “operational trigger threshold” based on tick density quartiles or deciles. This is an interesting suggestion but remains hypothetical. Please rephrase to avoid overinterpretation, or add a brief caveat that such thresholds require field validation.**

**Authors’ response:** We thank the reviewer for this important comment. We agree that the proposed "trigger thresholds" are exploratory and have not been validated as transmission thresholds. In addition, following suggestions from other reviewers, we have revised the relevant discussion to elaborate on the implications of our findings for tick-borne diseases. In the revised manuscript, we emphasize that while the tick ecological suitability identified in this study does not provide direct evidence linking it to SFTS or *Bandavirus dabieense* circulation, it remains a foundational component within the One Health framework. Future research should build on this ecological baseline by integrating tick infection testing, animal serology, and spatially resolved human case data to establish a more comprehensive virus–vector–host–human framework for SFTS risk assessment.

**The revised text in Discussion now reads:**

“Our study did not include Bandavirus dabieense detection in ticks, serological evidence from animal hosts or humans, or spatially matched analyses with county-level SFTS incidence. Human SFTS occurrence depends not only on vector habitat suitability, but also on local tick density, virus circulation in tick-host networks, animal host communities, human exposure behavior, and surveillance sensitivity. Nevertheless, from a One Health perspective, this vector ecological layer remains important because tick surveillance is a fundamental component of zoonotic disease prevention. By identifying where dominant tick species are seasonally abundant and environmentally suitable, our findings may help prioritize locations for active tick collection, Bandavirus dabieense testing, host surveillance, and risk characterizing. Future studies should build on this ecological baseline by integrating tick infection testing, animal serology, and spatially resolved human case data to develop a more complete virus-vector-host-human framework for SFTS risk assessment and climate-sensitive early warning in eastern China, as such integrated evidence would be important for understanding Bandavirus dabieense circulation and its potential cross-regional spread”.

**9. Limitations:**

**The limitations section acknowledges spatial heterogeneity and the exclusive use of bioclimatic variables. Please also acknowledge that the MaxEnt model predicts habitat suitability, not actual tick presence or abundance, and that** **future projections** **assume no changes in land use or host availability.**

**Authors’ response:** We thank the reviewer for this important suggestion. We acknowledged that future projections assume static land use and host availability, which may introduce additional uncertainty into long-term predictions. We have also revised the limitations section to clarify that the MaxEnt model estimates climatic habitat suitability rather than observed tick presence or abundance.

**The revised text now reads:**

“The study had several limitations. First, tick surveillance data were spatially heterogeneous, with relatively limited occurrence records in some areas, which may introduce uncertainty into model predictions. Second, to ensure the feasibility of future projections, only bioclimatic variables from the WorldClim database were included, thereby excluding potentially time-varying socio-economic factors (e.g., land use and host availability) and assuming these conditions remain unchanged in future scenarios. Third, the MaxEnt model estimates potential habitat suitability results, which represent climate-driven habitat suitability rather than observed species presence or density”.

**10. Typographical / formatting errors –**

**Page 12, line “1 1 1 1...” – appears to be a formatting artifact from the PDF conversion. Please clean up the final manuscript to remove these repeated numbers.**

**Authors’ response:** Thank you for this important comment. We will verify the layout upon resubmission to avoid similar formatting issues.

**In the Abstract, “2.854 ticks/100 m” – use unspaced “/” consistently; also “336.5%” and “640.3%” – space before “%” is usually not used in English; check journal style.**

**Authors’ response:** Thank you for this important comment. We have revised the manuscript to ensure consistency in unit formatting, including standardized use of “/” in unit expressions and removal of unnecessary spaces before “%”.

**Figure 1 legend: the source URL is included – ensure that the map permission statement (GS (2024) 0650) complies with PLOS NTDs requirements.**

**Authors’ response:** Thank you for this important comment. We will keep in touch with the editor to provide the necessary materials for map licensing and comply with PLoS NTDs requirements.

**Reviewer #4:**

**The authors have conducted a thorough investigation of tick species composition, seasonal dynamics, habitat preferences, and climate-driven habitat suitability for two dominant tick species (Haemaphysalis longicornis and H. flava) in Jiangsu Province, China, using a six-year surveillance dataset (2019–2024) and an optimized MaxEnt modeling framework.**

**While these aspects fall under the journal's broad scope of “vector biology” and “demographic, ecological and social determinants”, the critical requirement for publication in PLOS Neglected Tropical Diseases is that all such analyses must be explicitly and directly linked to a neglected tropical disease – in this case, severe fever with thrombocytopenia syndrome (SFTS). Unfortunately, the manuscript contains no virological, serological, or epidemiological data on SFTS virus (SFTSV) or any other tick-borne pathogen. Specifically:**

**No detection of SFTSV RNA in collected ticks.**

**No data on tick infection rates (minimum infection rate, prevalence).**

**No serological evidence from reservoir hosts (e.g., livestock, wildlife) or humans.**

**No spatial or temporal correlation analysis between tick density/distribution and human SFTS cases at the county or city level.**

**The only connection to SFTS is made in the introduction and discussion sections, where the authors qualitatively claim that their tick habitat suitability maps overlap with previously reported SFTS case distributions. The results section itself contains no analysis that directly relates tick surveillance findings to any measure of disease burden or pathogen presence.**

**While the ecological modeling is technically sound and the long-term surveillance data are valuable, the manuscript in its current form addresses purely vector ecology and climate change biology without the necessary disease component. Publishing this work in PLOS NTD would misalign with the journal's core mission, which requires that the research advances understanding, prevention, or control of a specific neglected tropical disease – not just its vector.**

**The authors are encouraged to either to add a direct disease link by testing the collected ticks for SFTSV (e.g., using RT-PCR) and/or performing spatial modeling of infected tick density or human case risk; or the study is more appropriate to be submitted to a more appropriate journal focused on vector ecology, acarology, or climate-change biology.**

**Authors’ response:** We thank the reviewer for this careful and important assessment. We agree that the disease relevance of a vector ecological study submitted to PLOS Neglected Tropical Diseases should be made explicit and supported within the manuscript. In response, we have added a new Results subsection entitled “Spatial concordance between publicly reported SFTS burden and projected tick suitability” and a new supplementary table (Table S4) to link our vector habitat suitability analysis with publicly reported SFTS burden across the 13 prefecture-level cities in Jiangsu Province.

Specifically, we summarized published provincial surveillance data showing that 914 SFTS cases were reported in Jiangsu during 2014–2023, involving 49 counties. At the prefecture level, Nanjing and Huai’an were the two major endemic regions, with 489 and 236 reported cases, respectively, together accounting for 79.32% of all reported cases, whereas Nantong was the only prefecture-level city without reported SFTS cases. We then compared these city-level epidemiological patterns with the predicted habitat suitability indices for the two dominant tick species generated in the present study. The Results now state that Nanjing showed high habitat suitability for both H. longicornis and H. flava, southern Huai’an also exhibited high suitability for both dominant species, and cities with lower reported SFTS burdens generally corresponded to moderate or low predicted tick suitability.

We emphasize that this added analysis is intended to provide disease-relevant ecological context and to assess spatial concordance between reported SFTS burden and dominant tick habitat suitability. It is not presented as direct evidence of Bandavirus dabieense circulation, infected tick density, host exposure, or human infection risk. We have therefore revised the Discussion to make this distinction explicit. The revised text acknowledges that our study did not include Bandavirus dabieense detection in ticks, serological evidence from animal hosts or humans, or spatially matched analyses with county-level SFTS incidence.

At the same time, we respectfully emphasize that vector ecology remains an important component of One Health surveillance for tick-borne zoonoses. Because tick bites are a major route of human SFTS infection, identifying where dominant tick species are seasonally abundant and environmentally suitable provides a necessary ecological layer for targeted surveillance. We have revised the Discussion to explain that these suitability maps should be interpreted as ecological priority maps for active tick collection, Bandavirus dabieense testing, host surveillance, and future SFTS-oriented field investigations.

We sincerely thank the reviewer for identifying this point in the original manuscript. The revised manuscript now includes a Results-level disease-context analysis and a clearer Discussion of both the relevance and the limits of our findings. Future studies should build on this ecological baseline by integrating tick infection testing, animal serology, and spatially resolved human case data to develop a more complete virus-vector-host-human framework for SFTS risk assessment and climate-sensitive early warning in eastern China.

We sincerely thank the reviewer again for the careful assessment and constructive suggestions.

**The revised text:**

**Results**

**Spatial concordance between publicly reported SFTS burden and projected tick suitability**

To provide a disease-relevant ecological context for the vector distribution analysis, we summarized publicly reported SFTS burden across the 13 prefecture-level cities in Jiangsu Province and linked these epidemiological data with the predicted city-level tick habitat suitability in the present study (Table S4). Published provincial surveillance data reported 914 SFTS cases in Jiangsu during 2014~2023, involving 49 counties. Nanjing and Huai’an were the two major endemic regions, with 489 and 236 reported cases, respectively, together accounting for 79.32% of all cases, whereas Nantong was the only prefecture-level city without reported SFTS cases. The remaining ten affected cities together accounted for 189 cases and were therefore classified as reported low-burden cities. The city-level comparison suggested a broad spatial concordance between reported SFTS burden and predicted habitat suitability for the two dominant tick species, particularly in major endemic areas such as Nanjing and Huai’an. In Nanjing, which had a high SFTS burden, both H. longicornis and H. flava showed high suitability indices. Similarly, southern Huai’an also exhibited high suitability indices for both dominant tick species. For several other cities with lower reported SFTS burdens, predicted tick suitability was generally moderate or low, although city-level concordance was not expected to be perfect because human SFTS occurrence also depends on virus circulation, host ecology, human exposure, and surveillance sensitivity.

**Discussion**

The high-suitability areas identified for the two dominant tick species in this study were concentrated mainly in the southwestern hilly region of Jiangsu, a pattern that was broadly concordant with independently reported SFTS epidemiology in the province. Tao et al. [22] analyzed notified SFTS cases from 2014 to 2023 and reported 914 cases across 49 counties in Jiangsu. At the prefecture level, Nanjing and Huai’an were the two major endemic regions, with 489 and 236 reported cases, respectively, together accounting for 79.32% of all reported cases, whereas Nantong had no reported SFTS cases. This distribution was generally consistent with our city-level MaxEnt predictions. Nanjing showed high habitat suitability for both *H. longicornis* and H. flava, and southern Huai’an also exhibited high suitability for the two dominant species. In contrast, the remaining affected cities, which had much lower reported case numbers, generally corresponded to moderate or low predicted tick suitability.

This spatial concordance suggests that the low mountain and hilly landscapes, forest-grass ecotones, and rural environments in southwestern Jiangsu may provide ecological conditions favorable for dominant tick populations and may also help explain the spatial concentration of reported SFTS cases. However, this concordance should be interpreted as disease-relevant ecological context rather than direct evidence of *Bandavirus dabieense* circulation or human disease risk. Our study did not include *Bandavirus dabieense* detection in ticks, serological evidence from animal hosts or humans, or spatially matched analyses with county-level SFTS incidence. Human SFTS occurrence depends not only on vector habitat suitability, but also on local tick density, virus circulation in tick-host networks, animal host communities, human exposure behavior, and surveillance sensitivity.

Nevertheless, from a One Health perspective, this vector ecological layer remains important because tick surveillance is a fundamental component of zoonotic disease prevention. By identifying where dominant tick species are seasonally abundant and environmentally suitable, our findings may help prioritize locations for active tick collection, *Bandavirus dabieense* testing, host surveillance, and risk characterization. Future studies should build on this ecological baseline by integrating tick infection testing, animal serology, and spatially resolved human case data to develop a more complete virus-vector-host-human framework for SFTS risk assessment and climate-sensitive early warning in eastern China, as such integrated evidence would be important for understanding *Bandavirus dabieense* circulation and its potential cross-regional spread.

**Table S4.** Spatial concordance between publicly reported SFTS burden and predicted tick suitability

| **Prefecture-level city** | **Publicly reported case evidence** | **Published quantitative / spatial detail** | **SFTS burden category** | **Predicted habitat suitability of H. longicornis under current climatic conditions** | **Predicted habitat suitability of H. flava under current climatic conditions** | **Source URLs** |
| --- | --- | --- | --- | --- | --- | --- |
| Nanjing | 489 cases reported during 2014-2023 | Major endemic region; Lishui County 106 cases and Jiangning County 51 cases during 2011-2021; Lishui had CFR 17.2% (23/134) among 2011-2022 fatal-case analysis; two urban SFTS cases reported in Nanjing in 2025. | High-burden city | High suitability across whole city | High suitability across whole city | https://www.frontiersin.org/journals/public-health/articles/10.3389/fpubh.2025.1662670/full; https://link.springer.com/article/10.1186/s12889-023-15379-3; https://www.frontiersin.org/journals/public-health/articles/10.3389/fpubh.2023.1076226/full; https://weekly.chinacdc.cn/article/doi/10.46234/ccdcw2026.040 |
| Huai'an | 236 cases reported during 2014-2023; 337 cases during 2011-2024 in Huai'an-specific study | Major endemic region; Xuyi County 332/337 Huai'an cases (98.52%) and all Huai'an deaths during 2011-2024; Xuyi County 146 cases during 2011-2021; most likely space-time cluster, RR=8.18. | High-burden city | High suitability in southern region and unsuitable in northern region | High suitability in southern region and unsuitable in northern region | https://www.frontiersin.org/journals/public-health/articles/10.3389/fpubh.2025.1662670/full; https://link.springer.com/article/10.1186/s12889-023-15379-3; https://www.zjyfyxzz.com/CN/10.19485/j.cnki.issn2096-5087.2026.01.010 |
| Zhenjiang | Exact city count not separately reported; included among 12 affected cities during 2011-2021 and 2014-2023; part of remaining 189 cases outside Nanjing and Huai'an in 2014-2023 | BMC Public Health analysis described cases as mainly distributed in Nanjing, Huai'an and Zhenjiang. | Moderate / cluster-associated | High and moderate suitability across whole city | High suitability across whole city | https://www.frontiersin.org/journals/public-health/articles/10.3389/fpubh.2025.1662670/full; https://link.springer.com/article/10.1186/s12889-023-15379-3 |
| Changzhou | Exact city count not separately reported; reported affected city; part of remaining 189 cases outside Nanjing and Huai'an in 2014-2023 | Liyang County was included in local High-High cluster areas during 2011-2021. | Moderate / cluster-associated | High and moderate suitability across whole city | High suitability in southern region | https://www.frontiersin.org/journals/public-health/articles/10.3389/fpubh.2025.1662670/full; https://link.springer.com/article/10.1186/s12889-023-15379-3 |
| Wuxi | Exact city count not separately reported in 2014-2023 provincial study; part of remaining 189 cases outside Nanjing and Huai'an; historical Yixing cluster of 11 patients in 1996 | Yixing County has historical SFTS-like cluster evidence and was mentioned among western/southern hilly fatal-case areas in Jiangsu. | Low-burden | Moderate and low suitability across whole city | Moderate and low suitability across whole city | https://www.frontiersin.org/journals/public-health/articles/10.3389/fpubh.2025.1662670/full; https://www.frontiersin.org/journals/public-health/articles/10.3389/fpubh.2023.1076226/full; https://journals.plos.org/plosntds/article?id=10.1371/journal.pntd.0006603 |
| Lianyungang | Exact city count not separately reported; reported affected city; part of remaining 189 cases outside Nanjing and Huai'an in 2014-2023 | Haizhou County was mentioned among western/southern hilly areas with fatal cases in the 2011-2022 fatal-case study. | Low-burden | Low suitability and unsuitable across whole city | Low suitability and unsuitable across whole city | https://www.frontiersin.org/journals/public-health/articles/10.3389/fpubh.2025.1662670/full |
| Yancheng | Exact city count not separately reported; reported affected city; part of remaining 189 cases outside Nanjing and Huai'an in 2014-2023 | No city-specific public count identified in the main provincial publication; classify as reported lower-burden relative to Nanjing and Huai'an. | Low-burden | Unsuitable across whole city | Moderate and low suitability across whole city | https://www.frontiersin.org/journals/public-health/articles/10.3389/fpubh.2025.1662670/full |
| Yangzhou | Exact city count not separately reported; reported affected city; part of remaining 189 cases outside Nanjing and Huai'an in 2014-2023 | No city-specific public count identified in the main provincial publication; classify as reported lower-burden relative to Nanjing and Huai'an. | Low-burden | High suitability in southern region and unsuitable in most region | High suitability in southern region and moderate in most region | https://www.frontiersin.org/journals/public-health/articles/10.3389/fpubh.2025.1662670/full |
| Taizhou | Exact city count not separately reported; reported affected city; part of remaining 189 cases outside Nanjing and Huai'an in 2014-2023 | No city-specific public count identified in the main provincial publication; classify as reported lower-burden relative to Nanjing and Huai'an. | Low-burden | Unsuitable across whole city | Moderate suitability across whole city | https://www.frontiersin.org/journals/public-health/articles/10.3389/fpubh.2025.1662670/full |
| Suzhou | Exact city count not separately reported; reported affected city; part of remaining 189 cases outside Nanjing and Huai'an in 2014-2023 | No city-specific public count identified in the main provincial publication; classify as reported lower-burden relative to Nanjing and Huai'an. | Low-burden | Moderate suitability and unsuitable across whole city | Moderate suitability across whole city | https://www.frontiersin.org/journals/public-health/articles/10.3389/fpubh.2025.1662670/full |
| Xuzhou | Exact city count not separately reported; reported affected city; part of remaining 189 cases outside Nanjing and Huai'an in 2014-2023 | No city-specific public count identified in the main provincial publication; classify as reported lower-burden relative to Nanjing and Huai'an. | Low-burden | Moderate suitability and unsuitable across whole city | Unsuitable across whole city | https://www.frontiersin.org/journals/public-health/articles/10.3389/fpubh.2025.1662670/full |
| Suqian | Exact city count not separately reported; reported affected city; part of remaining 189 cases outside Nanjing and Huai'an in 2014-2023 | No city-specific public count identified in the main provincial publication; classify as reported lower-burden relative to Nanjing and Huai'an. | Low-burden | Low suitability and unsuitable across whole city | Moderate and low suitability and e across whole city | https://www.frontiersin.org/journals/public-health/articles/10.3389/fpubh.2025.1662670/full |
| Nantong | 0 cases reported in 2014-2023 provincial study | Only prefecture-level city without reported SFTS cases in the 2014-2023 provincial epidemiological study. | Low-burden: No reported cases | Unsuitable across whole city | Moderate suitability across whole city | https://www.frontiersin.org/journals/public-health/articles/10.3389/fpubh.2025.1662670/full |
