# Supplementary material for: Spatial and seasonal patterns of tick distribution and climate-driven shifts in Jiangsu Province, an SFTS-affected region of eastern China
Source: PLoS Negl Trop Dis. 2026 Nov 4;20(11):e0014847. doi: 10.1371/journal.pntd.0014847 (PMC13754229; doi:10.1371/journal.pntd.0014847)
Supplement: Supplementary file 3 [file pntd.0014847.s011.docx]

**Response to Reviewers for PNTD-D-26-00682R1**

**Dear Editors and Reviewers,**

We would like to sincerely thank you for taking the time to review our manuscript and for providing thoughtful and constructive comments. We truly appreciate the opportunity to revise our manuscript for consideration in this journal. We found the Reviewers’ comments and suggestions enormously helpful in improving the quality and clarity of our work. We have carefully addressed all comments and revised the manuscript accordingly. Detailed point-by-point responses are provided below.

**Response to Reviewers**

Reviewer #2:

**Summary and General Comments**

The manuscript had been improved after the major revision with point to point responses for reviewer's comments. Minor revision might be recommended to the article.

**Authors’ response:** We sincerely thank Reviewer #2 for the positive assessment of our revised manuscript. We appreciate the suggestion and have carefully checked the manuscript once more to improve the clarity and accuracy of the presentation.

**Methods**

The objectives of the study had been clearly articulated with a clear testable hypothesis stated.

The study design is appropriate to address the stated objectives.

The population had been clearly described and appropriate for the hypothesis being tested.

The sample size appeared sufficient to ensure adequate power to address the hypothesis being tested.

Correct statistical analysis had been applied to support conclusions.

All there concerns about ethical or regulatory requirements met well to the Journal.

**Authors’ response:** We are thankful to Reviewer #2 for the supportive comments and positive assessment of **Methods**.

**Results**

The analysis presented match the analysis plan well.

All the results had been clearly and completely presented.

All the figures (Tables, Images and supplemental materials) had been demonstrated with sufficient quality for clarity.

**Authors’ response:** We sincerely thank Reviewer #2 for the positive evaluation of **Results**.

**Conclusions**

The conclusions was well supported by the data presented.

The limitations of analysis had been clearly described in the section of discussion

The authors discussed how these data can be helpful to advance our understanding of the topic under study.

The content of the research were presented with public health relevance.

**Authors’ response:** We sincerely thank Reviewer #2 for the positive evaluation of **Conclusions**.

**Editorial and Data Presentation Modifications**

The manuscript had been improved after the major revision. However, there are still minor revision needed before be accepted for publication.

1. Although the research mainly focused the territory of Jiangsu Province, the title “…affected regions of eastern China” might be beyond the content.

**Authors’ response:** We sincerely thank the reviewer for the insightful comment. We agree that the original title might have overstated the geographic scope of our study. To more accurately reflect the study area, we revised the title to “Spatial and seasonal patterns of tick distribution and climate-driven shifts in Jiangsu Province, an *SFTS*-affected region of eastern China.” We believe this revised version better represents the content of the manuscript.

2. In the section of Introduction, the expression of “In China, SFTS cases have been reported in multiple provinces, with affected areas predominantly in central and eastern regions” should be clarified and reorganized.

**Authors’ response:** We thank the reviewer for this helpful comment. We have revised the sentence in the **Introduction** to clarify and reorganize the description of the geographical distribution of SFTS cases in China. The revised sentence now reads: “In China, SFTS cases have been documented in multiple provinces. However, the affected areas are predominantly concentrated in the central and eastern regions. This geographical clustering of cases is closely associated with the regional distribution of tick vectors [18–20].” This revision explicitly links the spatial concentration of cases to the ecological distribution of tick vectors, thereby providing a clearer rationale for the subsequent discussion of our study area: Jiangsu Province.

3. In the section of Introduction, “Monitoring of free-living ticks in Yangzhou City from 2020 to 2024 found H. longicornis (70.01%) and H. flava (29.99%) as the only species detected [25]”, which was targeted as the only species?

**Authors’ response:** We apologize for the ambiguity. We intended to state that only two tick species were detected in the Yangzhou surveillance study, namely H. longicornis and H. flava.

We have revised the sentence accordingly:

“Monitoring of free-living ticks in Yangzhou City from 2020 to 2024 detected only two tick species, H. longicornis and H. flava, accounting for 70.01% and 29.99% of collected ticks, respectively”

4. In the section of Results, is any correlation between the tick density and surveillance events indicating by Figure 3? The clear explanation would be appreciated in the revised version of the manuscript.

**Authors’ response:** We thank the reviewer for this important comment. We added a correlation analysis between the tick density and surveillance events involving all tick species. Tick density was positively correlated with surveillance events (Pearson r = 0.785, *P* = 0.012; Spearman ρ = 0.883, *P* = 0.002), suggesting that sampling effort may have contributed to the observed seasonal distribution to some extent. We have therefore added this information to the **Methods**, **Results** and **Discussion**.

We acknowledge that the observed seasonal pattern may have been partly influenced by month-to-month variation in surveillance events. Nevertheless, the present dataset still provides a meaningful provincial-level view of tick dynamics and an important basis for further investigation. In future work, we will take these factors into account and further explore the seasonal characteristics of ticks based on the present study.

The revised **Methods**: “To examine whether monthly surveillance intensity covaried with observed tick density, Pearson and Spearman correlation coefficients were calculated between the monthly number of surveillance events and monthly mean total tick density across all tick species.”

The revised **Results**: “…Notably, monthly surveillance events were positively correlated with monthly total tick density (Pearson r = 0.785, *P* = 0.012; Spearman ρ = 0.883, *P* = 0.002), suggesting that sampling effort may have influenced the observed seasonal pattern to some extent.”

The revised **Discussion**: “In this study, we observed seasonal variation in monthly tick density for both *H. longicornis* and *H. flava*, with post-larval ticks concentrated in spring, particularly in April, and larvae peaking in autumn, particularly in September….... However, the positive correlation between tick density and surveillance events suggests that the observed seasonal distribution may partly reflect variation in sampling intensity across months. Even so, the provincial-level seasonal pattern identified in this study remains informative and provides a useful basis for future work aimed at further characterizing tick seasonal distribution.”

“The study had several limitations. First, tick surveillance data were temporally and spatially heterogeneous, with more surveillance events in April, May and September, and with relatively limited occurrence records in some areas. Therefore, the observed seasonal distribution may partly reflect sampling bias, and such heterogeneity may introduce uncertainty into model predictions.”

We sincerely thank the reviewer again for the careful assessment and constructive suggestions.

5. Ensure all the scientific name including the abbreviation be spelled in italic style.

**Authors’ response:** We thank the reviewer for the suggestion. We have carefully checked the scientific names of the biological species and have ensured that these names, as well as their abbreviations, are correctly presented in italic format throughout the manuscript.

Reviewer #3:

**Summary and General Comments**

The manuscript now presents a well-conducted, province-scale integration of long-term field surveillance and optimized ecological niche modeling, with clear relevance to SFTS prevention within a One Health framework. The revisions have satisfactorily addressed my previous concerns. I support publication of this work in PLOS Neglected Tropical Diseases.

**Authors’ response:** We sincerely thank Reviewer #3 for the thoughtful and encouraging evaluation of our revised manuscript. We appreciate the recognition of our efforts in addressing the previous concerns, and we are grateful for the invaluable suggestions that have helped improve the overall quality of the work. Thanks again for the time and support.

**Methods**

The authors have significantly clarified the sentinel site sampling strategy, survey frequency, and the rationale for using presence-only MaxEnt modeling with 10,000 background points. The spatial thinning procedure (10 km buffer) and model tuning details (RM ranges, feature classes, AUC with standard deviations) are now clearly documented, enhancing reproducibility.

**Authors’ response:** We are thankful to Reviewer #3 for the supportive comments and positive assessment of our revised **Methods**.

**Results**

Figure 3 has been expanded to show species- and stage-specific seasonal dynamics (larval vs. post-larval), which strengthens the ecological interpretation and addresses concerns about data consistency.

Figure 4 now includes response curves for all environmental variables included in each model, improving transparency.

The addition of the new Results subsection on "Spatial concordance between publicly reported SFTS burden and projected tick suitability" (together with Table S4) effectively addresses the critical concern raised by Reviewer #4 regarding the disease relevance of the study. While the authors acknowledge this is ecological context rather than direct pathogen evidence, the spatial linkage to reported SFTS cases in Nanjing and Huai'an provides meaningful public health grounding for PLOS NTDs.

**Authors’ response:** We are thankful to Reviewer #3 for the supportive comments and positive assessment of our revised **Results**.

**Conclusions**

(No Response)

**Editorial and Data Presentation Modifications**

Accept
