# Supplementary material for: Spatial and seasonal patterns of tick distribution and climate-driven shifts in Jiangsu Province, an SFTS-affected region of eastern China
Source: PLoS Negl Trop Dis. 2026 Nov 4;20(11):e0014847. doi: 10.1371/journal.pntd.0014847 (PMC13754229; doi:10.1371/journal.pntd.0014847)
Supplement: Supplementary file 4 [file pntd.0014847.s012.docx]

**Response to Reviewers for PNTD-D-26-00682R2**

**Dear Editors and Reviewers,**

We would like to sincerely thank you for taking the time to review our manuscript and for providing thoughtful and constructive comments. We truly appreciate the opportunity to revise our manuscript for consideration in this journal. We found the Reviewers’ comments and suggestions enormously helpful in improving the quality and clarity of our work. We have carefully addressed all comments and revised the manuscript accordingly. Detailed point-by-point responses are provided below.

**Response to Reviewers**

**Additional Editor Comments (if provided):**

The article is almost ready to be accepted. But what concerns me is the authenticity of the data. It is difficult to distinguish between species of *Haemaphysalis* ticks, such as the *H. longicornis* and *H. flava*, especially for the larvae and nymphs. The authors declared the species level identification was based on morphological characteristics by trained personnel. To ensure the reliability of the data, we hope the author can provide the original pictures that distinguish between species of *Haemaphysalis* ticks, especially for the nymphs and larvae. Detailed descriptions are also necessary. The pictures can be provided as additional files.

**Authors’ response:**

Thank you for highlighting the importance of reliable species identification. We agree that photographic documentation and explicit descriptions of the distinguishing characters are needed to make our identification procedure more transparent.

In response, we have provided original ventral micrographs of *H. longicornis* and *H. flava* nymphs and larvae in Supporting Information (**S2 Figure and S3 Figure**). The nymphal images show the four pairs of legs and the absence of a genital aperture, as well as the species-specific morphology of the ventral spur on palpal segment III. The larval images show the three pairs of legs and the morphology of the internal spurs of coxae I–III. The relevant morphological features are described in the accompanying figure legends, with enlarged views provided where appropriate.

We have also added a brief description to the Methods section. The corresponding photographs and morphological descriptions are provided in **S2 Figure** and **S3 Figure**.

These additions provide direct visual documentation of the morphological features used to distinguish *H. longicornis* from *H. flava*, with particular emphasis on the nymphal and larval stages.

The revised **Methods**:

“Specimens were transported to the laboratory for species-level identification based on morphological characteristics by trained personnel. Representative original ventral micrographs of *H. longicornis* and *H. flava* nymphs and larvae are provided in S2 Figure and S3 Figure.”

The revised **Supporting Information**:

**S2 Figure.** Morphological Microscopic Features of *Haemaphysalis longicornis* and *Haemaphysalis flava* Nymphs.

* Panel A: Ventral view of *H. longicornis* nymph (magnification: 90×). Panel B: Ventral view of *H. flava* nymph (magnification: 100×). Scale bar: 500 μm.

Both nymphs possess four pairs of legs and lack a genital pore. The *H. longicornis* nymph (panel A) bears a conical ventral spur on palpal segment III, with its tip reaching approximately the midpoint of palpal segment II. In the *H. flava* nymph (panel B), the ventral spur on palpal segment III is very short, stout and inconspicuous.

**Figure S3.** Morphological features of *Haemaphysalis longicornis* and *Haemaphysalis flava* larvae.

* Panel A: Ventral view of *H. longicornis* larva (magnification, 200×). Panel B: Ventral view of *H. flava* larva (magnification, 200×). Scale bar: 200 μm.

Both larvae possess three pairs of legs. The *H. longicornis* larva (panel A) exhibits a short, stout, triangular internal spur on coxa I with a pointed tip. The internal spurs on coxae II and III are somewhat ridge-like, with the spur on coxa III being indistinct. The *H. flava* larva (panel B) exhibits broad and short internal spurs on coxae II and III.
