# Supplementary material for: Spatial and seasonal patterns of tick distribution and climate-driven shifts in Jiangsu Province, an SFTS-affected region of eastern China
Source: PLoS Negl Trop Dis. 2026 Nov 4;20(11):e0014847. doi: 10.1371/journal.pntd.0014847 (PMC13754229; doi:10.1371/journal.pntd.0014847)
Supplement: Peer review history

# Peer review: Decision Letter 0

**DOI:** 10.1371/journal.pntd.0014847.r001

**Authors:** Amy Morrison, Yong Qi

11 May 2026

PNTD-D-26-00682

Spatiotemporal dynamics and climate-driven shifts of tick distribution in a SFTS-affected region of eastern China

PLOS Neglected Tropical Diseases

Dear Dr. Yao,

Thank you for submitting your manuscript to PLOS Neglected Tropical Diseases. After careful consideration, we feel that it has merit but does not fully meet PLOS Neglected Tropical Diseases's publication criteria as it currently stands. Therefore, we invite you to submit a revised version of the manuscript that addresses the points raised during the review process.

Please submit your revised manuscript within by Jul 10 2026 11:59PM. If you will need more time than this to complete your revisions, please reply to this message or contact the journal office at plosntds@plos.org. When you're ready to submit your revision, log on to https://www.editorialmanager.com/pntd/ and select the 'Submissions Needing Revision' folder to locate your manuscript file.

Please include the following items when submitting your revised manuscript:

* A letter that responds to each point raised by the editor and reviewer(s). You should upload this letter as a separate file labeled 'Response to Reviewers'. This file does not need to include responses to any formatting updates and technical items listed in the 'Journal Requirements' section below.

* A marked-up copy of your manuscript that highlights changes made to the original version. You should upload this as a separate file labeled 'Revised Manuscript with Track Changes'.

* An unmarked version of your revised paper without tracked changes. You should upload this as a separate file labeled 'Manuscript'.

If you would like to make changes to your financial disclosure, competing interests statement, or data availability statement, please make these updates within the submission form at the time of resubmission. Guidelines for resubmitting your figure files are available below the reviewer comments at the end of this letter.

As the corresponding author, your ORCID iD is verified in the submission system and will appear in the published article. PLOS supports the use of ORCID, and we encourage all coauthors to register for an ORCID iD and use it as well. Please encourage your coauthors to verify their ORCID iD within the submission system before final acceptance, as unverified ORCID iDs will not appear in the published article. Only the individual author can complete the verification step; PLOS staff cannot verify ORCID iDs on behalf of authors.

We look forward to receiving your revised manuscript.

Kind regards,

Yong Qi

Academic Editor

PLOS Neglected Tropical Diseases

Amy Morrison

Section Editor

PLOS Neglected Tropical Diseases

Shaden Kamhawi

co-Editor-in-Chief

PLOS Neglected Tropical Diseases

orcid.org/0000-0003-4304-636XX

Paul Brindley

co-Editor-in-Chief

PLOS Neglected Tropical Diseases

orcid.org/0000-0003-1765-0002

**Journal Requirements:**

If the reviewer comments include a recommendation to cite specific previously published works, please review and evaluate these publications to determine whether they are relevant and should be cited. There is no requirement to cite these works unless the editor has indicated otherwise.

1) Please provide an Author Summary. This should appear in your manuscript between the Abstract (if applicable) and the Introduction, and should be 150-200 words long. The aim should be to make your findings accessible to a wide audience that includes both scientists and non-scientists. Sample summaries can be found on our website under Submission Guidelines:

https://journals.plos.org/plosntds/s/submission-guidelines#loc-parts-of-a-submission

2) Your manuscript's sections are not in the correct order.  Please amend to the following order: Abstract, Introduction, Results, Discussion, and Methods

3) Please upload all main figures as separate Figure files in .tif or .eps format. For more information about how to convert and format your figure files please see our guidelines:

https://journals.plos.org/plosntds/s/figures

4) We have noticed that you have uploaded Supporting Information files, but you have not included a list of legends. Please add a full list of legends for your Supporting Information files after the references list.

5) Some material included in your submission may be copyrighted. According to PLOS’s copyright policy, authors who use figures or other material (e.g., graphics, clipart, maps) from another author or copyright holder must demonstrate or obtain permission to publish this material under the Creative Commons Attribution 4.0 International (CC BY 4.0) License used by PLOS journals. Please closely review the details of PLOS’s copyright requirements here: PLOS Licenses and Copyright. If you need to request permissions from a copyright holder, you may use PLOS's Copyright Content Permission form.

Please respond directly to this email and provide any known details concerning your material's license terms and permissions required for reuse, even if you have not yet obtained copyright permissions or are unsure of your material's copyright compatibility. Once you have responded and addressed all other outstanding technical requirements, you may resubmit your manuscript within Editorial Manager.

Potential Copyright Issues:

i) Figures 1, 2, and 5. Please (a) provide a direct link to the base layer of the map (i.e., the country or region border shape) and ensure this is also included in the figure legend; and (b) provide a link to the terms of use / license information for the base layer image or shapefile. We cannot publish proprietary or copyrighted maps (e.g. Google Maps, Mapquest) and the terms of use for your map base layer must be compatible with our CC BY 4.0 license.

Note: if you created the map in a software program like R or ArcGIS, please locate and indicate the source of the basemap shapefile onto which data has been plotted.

If your map was obtained from a copyrighted source please amend the figure so that the base map used is from an openly available source. Alternatively, please provide explicit written permission from the copyright holder granting you the right to publish the material under our CC BY 4.0 license.

If you are unsure whether you can use a map or not, please do reach out and we will be able to help you. The following websites are good examples of where you can source open access or public domain maps:

* U.S. Geological Survey (USGS) - All maps are in the public domain. (http://www.usgs.gov)

* PlaniGlobe - All maps are published under a Creative Commons license so please cite u201cPlaniGlobe, http://www.planiglobe.com, CC BY 2.0u201d in the image credit after the caption. (http://www.planiglobe.com/?lang=enl)

* Natural Earth - All maps are public domain. (http://www.naturalearthdata.com/about/terms-of-use/).

6) We note that you have indicated that there are restrictions to data sharing for this study. PLOS only allows data to be available upon request if there are legal or ethical restrictions on sharing data publicly. For more information on unacceptable data access restrictions, please see https://journals.plos.org/plosntds/s/data-availability#loc-unacceptable-data-access-restrictions.

Before we proceed with your manuscript, please address the following prompts:

a) If there are ethical or legal restrictions on sharing a de-identified data set, please explain them in detail (e.g., data contain potentially identifying or sensitive patient information, data are owned by a third-party organization, etc.) and who has imposed them (e.g., a Research Ethics Committee or Institutional Review Board, etc.). Please also provide contact information for a data access committee, ethics committee, or other institutional body to which data requests may be sent.

b) If there are no restrictions, please upload the minimal anonymized data set necessary to replicate your study findings to a stable, public repository and provide us with the relevant URLs, DOIs, or accession numbers. For a list of recommended repositories, please see https://journals.plos.org/plosone/s/recommended-repositories. You also have the option of uploading the data as Supporting Information files, but we would recommend depositing data directly to a data repository if possible.

We will update your Data Availability statement on your behalf to reflect the information you provide.

7) Please amend your detailed Financial Disclosure statement. This is published with the article. It must therefore be completed in full sentences and contain the exact wording you wish to be published.

1) State the initials, alongside each funding source, of each author to receive each grant. For example: "This work was supported by the National Institutes of Health (####### to AM; ###### to CJ) and the National Science Foundation (###### to AM)."

2) State what role the funders took in the study. If the funders had no role in your study, please state: "The funders had no role in study design, data collection and analysis, decision to publish, or preparation of the manuscript."

3) If any authors received a salary from any of your funders, please state which authors and which funders..

If you did not receive any funding for this study, please simply state: u201cThe authors received no specific funding for this work.u201d

**Reviewers' Comments:**

Reviewer's Responses to Questions

**Key Review Criteria Required for Acceptance?**

As you describe the new analyses required for acceptance, please consider the following:

**Methods**

-Are the objectives of the study clearly articulated with a clear testable hypothesis stated?

-Is the study design appropriate to address the stated objectives?

-Is the population clearly described and appropriate for the hypothesis being tested?

-Is the sample size sufficient to ensure adequate power to address the hypothesis being tested?

-Were correct statistical analysis used to support conclusions?

-Are there concerns about ethical or regulatory requirements being met?

Reviewer #1: * The sampling strategy for sentinel sites and the survey frequency at each site should be clearly described.

* The method used to select absence (or pseudo-absence) locations for ENM should be specified.

* As an optional suggestion: given that multiple tick species are analyzed, a joint species distribution model could provide additional insights into potential interspecies interactions.

Reviewer #2: The objectives of the study had been clearly articulated with a clear testable hypothesis stated.

The study design needs to be updated to address the stated objectives.

The population has been clearly described and appropriate for the hypothesis being tested

The sample size is sufficient to ensure adequate power to address the hypothesis being tested, additional data on the parasitic ticks would be appreciated.

The correct statistical analysis had been applied to to support conclusions

The concerns about ethical or regulatory requirements have been met currently.

Reviewer #3: (No Response)

Reviewer #4: (No Response)

**********

**Results**

-Does the analysis presented match the analysis plan?

-Are the results clearly and completely presented?

-Are the figures (Tables, Images) of sufficient quality for clarity?

Reviewer #1: * The total number of sampling sites and the number of presence records for each species should be reported.

* Figure 2: Consider using point size or a color gradient to represent tick density across locations, which may also help validate ENM outputs.

* Figure 3: As the surveillance data are a key strength of this study, this figure could be expanded to include stratification by tick species and life stage. For example, H. longicornis typically shows an autumn peak corresponding to larvae, often with much higher densities than nymphs (spring) and adults (summer). The current pattern raises questions about data consistency or whether specific protocols were used for larval sampling.

* Figure 4: Only two response curves are shown, despite including seven environmental variables. It would be more informative to present all response curves.

* Figure 5: The classification criteria for “unsuitable,” “low,” “moderate,” and “high” suitability should be clarified. Additionally, panel (d) suggests that H. flava may be suitable in the southwest of Jiangsu, which appears inconsistent with Figure 1—this discrepancy should be explained.

Reviewer #2: The analysis presented basically match the analysis plan

The results ae clearly and completely presented.

The figures (Tables, Images) are presented with sufficient quality for clarity.

Reviewer #3: (No Response)

Reviewer #4: (No Response)

**********

**Conclusions**

-Are the conclusions supported by the data presented?

-Are the limitations of analysis clearly described?

-Do the authors discuss how these data can be helpful to advance our understanding of the topic under study?

-Is public health relevance addressed?

Reviewer #1: Title

* The title refers to “spatiotemporal dynamics,” but the manuscript primarily reports seasonality and spatial distribution, rather than true spatiotemporal dynamics. The title may need to be revised for accuracy.

Introduction

* Previous ENM studies of H. longicornis should be more comprehensively reviewed. The authors should clearly state the novelty of the current study relative to existing work and explicitly define the research gap being addressed. Relevant studies include, but are not limited to:

* Yang X, Gao Z, Wang L, Xiao L, Dong N, Wu H, Li S. Projecting the potential distribution of ticks in China under climate and land use change. International Journal for Parasitology. 2021 Aug 1;51(9):749-59.;

* Zhao GP, Wang YX, Fan ZW, Ji Y, Liu MJ, Zhang WH, Li XL, Zhou SX, Li H, Liang S, Liu W. Mapping ticks and tick-borne pathogens in China. Nature communications. 2021 Feb 17;12(1):1075.;

* Zhao L, Li J, Cui X, Jia N, Wei J, Xia L, Wang H, Zhou Y, Wang Q, Liu X, Yin C. Distribution of Haemaphysalis longicornis and associated pathogens: analysis of pooled data from a China field survey and global published data. The Lancet Planetary Health. 2020 Aug 1;4(8):e320-9.;

* Ding FY, Ge HH, Ma T, Wang Q, Hao MM, Li H, Zhang XA, Maude RJ, Wang LP, Jiang D, Fang LQ. Projecting spatiotemporal dynamics of severe fever with thrombocytopenia syndrome in the mainland of China. Global Change Biology. 2023 Dec;29(23):6647-60.

Discussion

* In paragraph 3, in addition to the two peaks discussed, there is typically a summer peak corresponding to adult activity, which should be acknowledged.

* The predicted suitability patterns and response curves should be compared more explicitly with findings from previous studies, highlighting consistencies or discrepancies.

Minor Comments

* Bandavirus dabieense (formerly Dabie bandavirus) should be italicized in the Introduction, and the updated nomenclature should be used consistently.

Reviewer #2: The conclusions are supported by the data presented.

The limitations of analysis needs to be updated to clearly described.

These data had been discussed to advance the understanding of the topic under current study.

The public health relevance had been addressed in the manuscript.

Reviewer #3: (No Response)

Reviewer #4: (No Response)

**********

**Editorial and Data Presentation Modifications?**

Use this section for editorial suggestions as well as relatively minor modifications of existing data that would enhance clarity. If the only modifications needed are minor and/or editorial, you may wish to recommend “Minor Revision” or “Accept”.

Reviewer #1: (No Response)

Reviewer #2: (No Response)

Reviewer #3: (No Response)

Reviewer #4: (No Response)

**********

**Summary and General Comments**

Use this section to provide overall comments, discuss strengths/weaknesses of the study, novelty, significance, general execution and scholarship. You may also include additional comments for the author, including concerns about dual publication, research ethics, or publication ethics. If requesting major revision, please articulate the new experiments that are needed.

Reviewer #1: This manuscript presents tick surveillance data from Jiangsu Province, one of the known hotspots for SFTS transmission in China, and models the potential distribution of H. longicornis and H. flava under future climate change scenarios. The tick surveillance dataset is novel and valuable, and could be further emphasized. While the ecological niche modeling (ENM) is also relevant, similar suitability mapping for these species has been conducted by multiple studies; therefore, the novelty of the methods and/or findings in the current study should be more clearly articulated.

Reviewer #2: In the manuscript, a provincial scale 5 years tick surveillance were performed in Jiangsu, Southeast China. Seasonal dynamics of tick communities and habitats-associated differences were characterized using an optimized MaxEnt modeling to reveal the determining environmental factors and hope to predict the potential health risk caused by ticks and tick-borne diseases. However, the current manuscript needs major revision before be accepted for publication.

1. Since the records of ticks and tick-borne diseases, especially for SFTS, in Jiangsu is paramount important to sponsor the current research, a brief and concise introduction of tick borne diseases in Jiangsu would be helpful.

2. The current surveillance had been performed targeting the off-host ticks, however, the parasitic ticks on domestical or wild animals would be highly recommended to be enrolled in the revised version.

3. As H. flava was described as one of the main targets, the references of the species on the transmission of SFTSV would be cited. Jang et al., 2024 Microbiol Spectr 12: e00489-24. Fang et al., 2023, Ticks and Tick-Borne Diseases 14, 102100

4. The Dabie Bandavirus had been proposed as “Bandavirus dabieense”,

Reviewer #3: (No Response)

Reviewer #4: The authors have conducted a thorough investigation of tick species composition, seasonal dynamics, habitat preferences, and climate-driven habitat suitability for two dominant tick species (Haemaphysalis longicornis and H. flava) in Jiangsu Province, China, using a six-year surveillance dataset (2019–2024) and an optimized MaxEnt modeling framework. While these aspects fall under the journal's broad scope of "vector biology" and "demographic, ecological and social determinants", the critical requirement for publication in PLOS Neglected Tropical Diseases is that all such analyses must be explicitly and directly linked to a neglected tropical disease – in this case, severe fever with thrombocytopenia syndrome (SFTS).

Unfortunately, the manuscript contains no virological, serological, or epidemiological data on SFTS virus (SFTSV) or any other tick-borne pathogen. Specifically:

No detection of SFTSV RNA in collected ticks.

No data on tick infection rates (minimum infection rate, prevalence).

No serological evidence from reservoir hosts (e.g., livestock, wildlife) or humans.

No spatial or temporal correlation analysis between tick density/distribution and human SFTS cases at the county or city level.

The only connection to SFTS is made in the introduction and discussion sections, where the authors qualitatively claim that their tick habitat suitability maps overlap with previously reported SFTS case distributions. The results section itself contains no analysis that directly relates tick surveillance findings to any measure of disease burden or pathogen presence.

While the ecological modeling is technically sound and the long-term surveillance data are valuable, the manuscript in its current form addresses purely vector ecology and climate change biology without the necessary disease component. Publishing this work in PLOS NTD would misalign with the journal's core mission, which requires that the research advances understanding, prevention, or control of a specific neglected tropical disease – not just its vector.

The authors are encouraged to either to add a direct disease link by testing the collected ticks for SFTSV (e.g., using RT-PCR) and/or performing spatial modeling of infected tick density or human case risk; or the study is more appropriate to be submitted to a more appropriate journal focused on vector ecology, acarology, or climate-change biology.

**********

PLOS authors have the option to publish the peer review history of their article (what does this mean?). If published, this will include your full peer review and any attached files.

If you choose “no”, your identity will remain anonymous but your review may still be made public.

**Do you want your identity to be public for this peer review?**  For information about this choice, including consent withdrawal, please see our Privacy Policy.

Reviewer #1: No

Reviewer #2: No

Reviewer #3: No

Reviewer #4: No

[NOTE: If reviewer comments were submitted as an attachment file, they will be attached to this email and accessible via the submission site. Please log into your account, locate the manuscript record, and check for the action link "View Attachments". If this link does not appear, there are no attachment files.]

**Figure resubmission:**

While revising your submission, we strongly recommend that you use PLOS’s NAAS tool (https://ngplosjournals.pagemajik.ai/artanalysis) to test your figure files. NAAS can convert your figure files to the TIFF file type and meet basic requirements (such as print size, resolution), or provide you with a report on issues that do not meet our requirements and that NAAS cannot fix.

After uploading your figures to PLOS’s NAAS tool - https://ngplosjournals.pagemajik.ai/artanalysis, NAAS will process the files provided and display the results in the "Uploaded Files" section of the page as the processing is complete. If the uploaded figures meet our requirements (or NAAS is able to fix the files to meet our requirements), the figure will be marked as "fixed" above. If NAAS is unable to fix the files, a red "failed" label will appear above. When NAAS has confirmed that the figure files meet our requirements, please download the file via the download option, and include these NAAS processed figure files when submitting your revised manuscript.

**Reproducibility:**

To enhance the reproducibility of your results, we recommend that authors of applicable studies deposit laboratory protocols in protocols.io, where a protocol can be assigned its own identifier (DOI) such that it can be cited independently in the future. Additionally, PLOS ONE offers an option to publish peer-reviewed clinical study protocols. Read more information on sharing protocols at https://plos.org/protocols?utm_medium=editorial-email&utm_source=authorletters&utm_campaign=protocols

Attachment Submitted filename: Comments for PNTD-D-26-00682.docx

# Author comment: Author response to Decision Letter 1

**DOI:** 10.1371/journal.pntd.0014847.r002

13 Jul 2026

Attachment Submitted filename: Response to Reviewers.docx

# Peer review: Decision Letter 1

**DOI:** 10.1371/journal.pntd.0014847.r003

**Authors:** Amy Morrison, Yong Qi

24 Aug 2026

PNTD-D-26-00682R1Spatial and seasonal patterns of tick distribution and climate-driven shifts in an SFTS-affected region of eastern ChinaPLOS Neglected Tropical DiseasesDear Dr. Yao,Thank you for submitting your manuscript to PLOS Neglected Tropical Diseases. After careful consideration, we feel that it has merit but does not fully meet PLOS Neglected Tropical Diseases's publication criteria as it currently stands. Therefore, we invite you to submit a revised version of the manuscript that addresses the points raised during the review process.Please submit your revised manuscript by Oct 23 2026 11:59PM. If you will need more time than this to complete your revisions, please reply to this message or contact the journal office at plosntds@plos.org. When you're ready to submit your revision, log on to https://www.editorialmanager.com/pntd/ and select the 'Submissions Needing Revision' folder to locate your manuscript file.Please include the following items when submitting your revised manuscript:* A letter that responds to each point raised by the editor and reviewer(s). You should upload this letter as a separate file labeled 'Response to Reviewers'. This file does not need to include responses to any formatting updates and technical items listed in the 'Journal Requirements' section below.* A marked-up copy of your manuscript that highlights changes made to the original version. You should upload this as a separate file labeled 'Revised Manuscript with Track Changes'.* An unmarked version of your revised paper without tracked changes. You should upload this as a separate file labeled 'Manuscript'.If you would like to make changes to your financial disclosure, competing interests statement, or data availability statement, please make these updates within the submission form at the time of resubmission. Guidelines for resubmitting your figure files are available below the reviewer comments at the end of this letter.As the corresponding author, your ORCID iD is verified in the submission system and will appear in the published article. PLOS supports the use of ORCID, and we encourage all coauthors to register for an ORCID iD and use it as well. Please encourage your coauthors to verify their ORCID iD within the submission system before final acceptance, as unverified ORCID iDs will not appear in the published article. Only the individual author can complete the verification step; PLOS staff cannot verify ORCID iDs on behalf of authors.We look forward to receiving your revised manuscript.Kind regards,Yong QiAcademic EditorPLOS Neglected Tropical DiseasesAmy MorrisonSection EditorPLOS Neglected Tropical Diseases

Shaden Kamhawi

co-Editor-in-Chief

PLOS Neglected Tropical Diseases

orcid.org/0000-0003-4304-636XX

Paul Brindley

co-Editor-in-Chief

PLOS Neglected Tropical Diseases

orcid.org/0000-0003-1765-0002

**Reviewers' comments:** Reviewer's Responses to Questions

**Key Review Criteria Required for Acceptance?**

As you describe the new analyses required for acceptance, please consider the following:

**Methods**

-Are the objectives of the study clearly articulated with a clear testable hypothesis stated?

-Is the study design appropriate to address the stated objectives?

-Is the population clearly described and appropriate for the hypothesis being tested?

-Is the sample size sufficient to ensure adequate power to address the hypothesis being tested?

-Were correct statistical analysis used to support conclusions?

-Are there concerns about ethical or regulatory requirements being met?

Reviewer #2: The objectives of the study had been clearly articulated with a clear testable hypothesis stated.

The study design is appropriate to address the stated objectives.

The population had been clearly described and appropriate for the hypothesis being tested.

The sample size appeared sufficient to ensure adequate power to address the hypothesis being tested.

Correct statistical analysis had been applied to support conclusions?

All there concerns about ethical or regulatory requirements met well to the Journal.

Reviewer #3: The authors have significantly clarified the sentinel site sampling strategy, survey frequency, and the rationale for using presence-only MaxEnt modeling with 10,000 background points. The spatial thinning procedure (10 km buffer) and model tuning details (RM ranges, feature classes, AUC with standard deviations) are now clearly documented, enhancing reproducibility.

**********

**Results**

-Does the analysis presented match the analysis plan?

-Are the results clearly and completely presented?

-Are the figures (Tables, Images) of sufficient quality for clarity?

Reviewer #2: The analysis presented match the analysis plan well.

All the results had been clearly and completely presented.

All the figures (Tables, Images and supplemental materials) had been demonstrated with sufficient quality for clarity.

Reviewer #3: Figure 3 has been expanded to show species- and stage-specific seasonal dynamics (larval vs. post-larval), which strengthens the ecological interpretation and addresses concerns about data consistency.

Figure 4 now includes response curves for all environmental variables included in each model, improving transparency.

The addition of the new Results subsection on "Spatial concordance between publicly reported SFTS burden and projected tick suitability" (together with Table S4) effectively addresses the critical concern raised by Reviewer #4 regarding the disease relevance of the study. While the authors acknowledge this is ecological context rather than direct pathogen evidence, the spatial linkage to reported SFTS cases in Nanjing and Huai'an provides meaningful public health grounding for PLOS NTDs.

**********

**Conclusions**

-Are the conclusions supported by the data presented?

-Are the limitations of analysis clearly described?

-Do the authors discuss how these data can be helpful to advance our understanding of the topic under study?

-Is public health relevance addressed?

Reviewer #2: The conclusions was well supported by the data presented.

The limitations of analysis had been clearly described in the section of discussion

The authors discussed how these data can be helpful to advance our understanding of the topic under study.

The content of the research were presented with public health relevance

Reviewer #3: (No Response)

**********

**Editorial and Data Presentation Modifications?**

Use this section for editorial suggestions as well as relatively minor modifications of existing data that would enhance clarity. If the only modifications needed are minor and/or editorial, you may wish to recommend “Minor Revision” or “Accept”.

Reviewer #2: The manuscript had been improved after the major revision. However, there are still minor revision needed before be accepted for publication.

1. Although the research mainly focused the territory of Jiangsu Province, the title “…affected regions of eastern China” might be beyond the content.

2. In the section of Introduction, the expression of “In China, SFTS cases have been reported in multiple provinces, with affected areas predominantly in central and eastern regions” should be clarified and reorganized.

3. In the section of Introduction, “Monitoring of freeliving ticks in Yangzhou City from 2020 to 2024 found H. longicornis (70.01%) and H. flava (29.99%) as the only species detected [25]”, which was targeted as the only species?

4. In the section of Results, is any correlation between the tick density and surveillance events indicating by Figure 3? The clear explanation would be appreciated in the revised version of the manuscript.

5. Ensure all the scientific name including the abbreviation be spelled in italic style.

Reviewer #3: Accept

**********

**Summary and General Comments**

Use this section to provide overall comments, discuss strengths/weaknesses of the study, novelty, significance, general execution and scholarship. You may also include additional comments for the author, including concerns about dual publication, research ethics, or publication ethics. If requesting major revision, please articulate the new experiments that are needed.

Reviewer #2: The manuscript had been improved after the major revision with point to point responses for reviewer's comments. Minor revision might be recommended to the article.

Reviewer #3: The manuscript now presents a well-conducted, province-scale integration of long-term field surveillance and optimized ecological niche modeling, with clear relevance to SFTS prevention within a One Health framework. The revisions have satisfactorily addressed my previous concerns. I support publication of this work in PLOS Neglected Tropical Diseases.

**********

PLOS authors have the option to publish the peer review history of their article (what does this mean?). If published, this will include your full peer review and any attached files.

If you choose “no”, your identity will remain anonymous but your review may still be made public.

**Do you want your identity to be public for this peer review?**  For information about this choice, including consent withdrawal, please see our Privacy Policy.

Reviewer #2: **Yes:**  Yi Sun

Reviewer #3: No

[NOTE: If reviewer comments were submitted as an attachment file, they will be attached to this email and accessible via the submission site. Please log into your account, locate the manuscript record, and check for the action link "View Attachments". If this link does not appear, there are no attachment files.] **Figure resubmission:**   While revising your submission, we strongly recommend that you use PLOS’s NAAS tool (https://ngplosjournals.pagemajik.ai/artanalysis) to test your figure files. NAAS can convert your figure files to the TIFF file type and meet basic requirements (such as print size, resolution), or provide you with a report on issues that do not meet our requirements and that NAAS cannot fix.

After uploading your figures to PLOS’s NAAS tool - https://ngplosjournals.pagemajik.ai/artanalysis, NAAS will process the files provided and display the results in the "Uploaded Files" section of the page as the processing is complete. If the uploaded figures meet our requirements (or NAAS is able to fix the files to meet our requirements), the figure will be marked as "fixed" above. If NAAS is unable to fix the files, a red "failed" label will appear above. When NAAS has confirmed that the figure files meet our requirements, please download the file via the download option, and include these NAAS processed figure files when submitting your revised manuscript. **Reproducibility:**  To enhance the reproducibility of your results, we recommend that authors of applicable studies deposit laboratory protocols in protocols.io, where a protocol can be assigned its own identifier (DOI) such that it can be cited independently in the future. Additionally, PLOS ONE offers an option to publish peer-reviewed clinical study protocols. Read more information on sharing protocols at https://plos.org/protocols?utm_medium=editorial-email&utm_source=authorletters&utm_campaign=protocols

# Author comment: Author response to Decision Letter 2

**DOI:** 10.1371/journal.pntd.0014847.r004

6 Sep 2026

Attachment Submitted filename: Response_letter.docx

# Editor report: Decision Letter 2

**DOI:** 10.1371/journal.pntd.0014847.r005

**Authors:** Amy Morrison, Yong Qi

5 Oct 2026

PNTD-D-26-00682R2

Spatial and seasonal patterns of tick distribution and climate-driven shifts in Jiangsu Province, an SFTS-affected region of eastern ChinaPLOS Neglected Tropical DiseasesDear Dr. Yao,Thank you for submitting your manuscript to PLOS Neglected Tropical Diseases. After careful consideration, we feel that it has merit but does not fully meet PLOS Neglected Tropical Diseases's publication criteria as it currently stands. Therefore, we invite you to submit a revised version of the manuscript that addresses the points raised during the review process.Please submit your revised manuscript by Dec 04 2026 11:59PM. If you will need more time than this to complete your revisions, please reply to this message or contact the journal office at plosntds@plos.org. When you're ready to submit your revision, log on to https://www.editorialmanager.com/pntd/ and select the 'Submissions Needing Revision' folder to locate your manuscript file.Please include the following items when submitting your revised manuscript:* A letter that responds to each point raised by the editor and reviewer(s). You should upload this letter as a separate file labeled 'Response to Reviewers'. This file does not need to include responses to any formatting updates and technical items listed in the 'Journal Requirements' section below.* A marked-up copy of your manuscript that highlights changes made to the original version. You should upload this as a separate file labeled 'Revised Manuscript with Track Changes'.* An unmarked version of your revised paper without tracked changes. You should upload this as a separate file labeled 'Manuscript'.If you would like to make changes to your financial disclosure, competing interests statement, or data availability statement, please make these updates within the submission form at the time of resubmission. Guidelines for resubmitting your figure files are available below the reviewer comments at the end of this letter.

As the corresponding author, your ORCID iD is verified in the submission system and will appear in the published article. PLOS supports the use of ORCID, and we encourage all coauthors to register for an ORCID iD and use it as well. Please encourage your coauthors to verify their ORCID iD within the submission system before final acceptance, as unverified ORCID iDs will not appear in the published article. Only the individual author can complete the verification step; PLOS staff cannot verify ORCID iDs on behalf of authors.We look forward to receiving your revised manuscript.Kind regards,Yong QiAcademic EditorPLOS Neglected Tropical DiseasesAmy MorrisonSection EditorPLOS Neglected Tropical Diseases

Shaden Kamhawi

co-Editor-in-Chief

PLOS Neglected Tropical Diseases

orcid.org/0000-0003-4304-636XX

Paul Brindley

co-Editor-in-Chief

PLOS Neglected Tropical Diseases

orcid.org/0000-0003-1765-0002

**Additional Editor Comments (if provided):** The article is almost ready to be accepted. But what concerns me is the authenticity of the data. It is difficult to distinguish between species of Haemaphysalis ticks, such as the H. longicornis and H. flava, especially for the larvae and nymphs. The authors declared the species level identification was based on morphological characteristics by trained personnel. To ensure the reliability of the data, we hope the author can provide the original pictures that distinguish between species of Haemaphysalis ticks, especially for the nymphs and larvae. Detailed descriptions are also necessary. The pictures can be provided as additional files.**Journal Requirements:**

If the reviewer comments include a recommendation to cite specific previously published works, please review and evaluate these publications to determine whether they are relevant and should be cited. There is no requirement to cite these works unless the editor has indicated otherwise.

1) We have amended your Competing Interest statement to comply with journal style. We kindly ask that you double check the statement and let us know if anything is incorrect.

**Reviewers' comments:** [NOTE: If reviewer comments were submitted as an attachment file, they will be attached to this email and accessible via the submission site. Please log into your account, locate the manuscript record, and check for the action link "View Attachments". If this link does not appear, there are no attachment files.]**Figure resubmission:** While revising your submission, we strongly recommend that you use PLOS’s NAAS tool (https://ngplosjournals.pagemajik.ai/artanalysis) to test your figure files. NAAS can convert your figure files to the TIFF file type and meet basic requirements (such as print size, resolution), or provide you with a report on issues that do not meet our requirements and that NAAS cannot fix.

After uploading your figures to PLOS’s NAAS tool - https://ngplosjournals.pagemajik.ai/artanalysis, NAAS will process the files provided and display the results in the "Uploaded Files" section of the page as the processing is complete. If the uploaded figures meet our requirements (or NAAS is able to fix the files to meet our requirements), the figure will be marked as "fixed" above. If NAAS is unable to fix the files, a red "failed" label will appear above. When NAAS has confirmed that the figure files meet our requirements, please download the file via the download option, and include these NAAS processed figure files when submitting your revised manuscript.**Reproducibility:** To enhance the reproducibility of your results, we recommend that authors of applicable studies deposit laboratory protocols in protocols.io, where a protocol can be assigned its own identifier (DOI) such that it can be cited independently in the future. Additionally, PLOS ONE offers an option to publish peer-reviewed clinical study protocols. Read more information on sharing protocols at https://plos.org/protocols?utm_medium=editorial-email&utm_source=authorletters&utm_campaign=protocols

# Author comment: Author response to Decision Letter 3

**DOI:** 10.1371/journal.pntd.0014847.r006

17 Oct 2026

Attachment Submitted filename: Response_letter_auresp_3.docx

# Editor report: Decision Letter 3

**DOI:** 10.1371/journal.pntd.0014847.r007

**Authors:** Amy Morrison, Yong Qi

22 Oct 2026

Dear Dr. Yao,

We are pleased to inform you that your manuscript 'Spatial and seasonal patterns of tick distribution and climate-driven shifts in Jiangsu Province, an SFTS-affected region of eastern China' has been provisionally accepted for publication in PLOS Neglected Tropical Diseases.

Before your manuscript can be formally accepted you will need to complete some formatting changes, which you will receive in a follow up email. A member of our team will be in touch with a set of requests.

Please note that your manuscript will not be scheduled for publication until you have made the required changes, so a swift response is appreciated.

IMPORTANT: The editorial review process is now complete. PLOS will only permit corrections to spelling, formatting or significant scientific errors from this point onwards. Requests for major changes, or any which affect the scientific understanding of your work, will cause delays to the publication date of your manuscript.

Should you, your institution's press office or the journal office choose to press release your paper, you will automatically be opted out of early publication. We ask that you notify us now if you or your institution is planning to press release the article. All press must be co-ordinated with PLOS.

Thank you again for supporting Open Access publishing; we are looking forward to publishing your work in PLOS Neglected Tropical Diseases.

Best regards,

Yong Qi

Academic Editor

PLOS Neglected Tropical Diseases

Amy Morrison

Section Editor

PLOS Neglected Tropical Diseases

Shaden Kamhawi

co-Editor-in-Chief

PLOS Neglected Tropical Diseases

orcid.org/0000-0003-4304-636XX

Paul Brindley

co-Editor-in-Chief

PLOS Neglected Tropical Diseases

orcid.org/0000-0003-1765-0002

***********************************************************

# Editor report: Acceptance letter

**DOI:** 10.1371/journal.pntd.0014847.r008

**Authors:** Amy Morrison, Yong Qi

Dear Dr. Yao,

We are delighted to inform you that your manuscript, "Spatial and seasonal patterns of tick distribution and climate-driven shifts in Jiangsu Province, an SFTS-affected region of eastern China," has been formally accepted for publication in PLOS Neglected Tropical Diseases.

We have now passed your article onto the PLOS Production Department who will complete the rest of the publication process. All authors will receive a confirmation email upon publication.

The corresponding author will soon be receiving a typeset proof for review, to ensure errors have not been introduced during production. Please review the PDF proof of your manuscript carefully, as this is the last chance to correct any scientific or type-setting errors. Please note that major changes, or those which affect the scientific understanding of the work, will likely cause delays to the publication date of your manuscript. Note: Proofs for Front Matter articles (Editorial, Viewpoint, Symposium, Review, etc...) are generated on a different schedule and may not be made available as quickly.

Soon after your final files are uploaded, the early version of your manuscript will be published online unless you opted out of this process. The date of the early version will be your article's publication date. The final article will be published to the same URL, and all versions of the paper will be accessible to readers.

For Research Articles, you will receive an invoice from PLOS for your publication fee after your manuscript has reached the completed accept phase. If you receive an email requesting payment before acceptance or for any other service, this may be a phishing scheme. Learn how to identify phishing emails and protect your accounts at https://explore.plos.org/phishing.

Thank you again for supporting open-access publishing; we are looking forward to publishing your work in PLOS Neglected Tropical Diseases.

Best regards,

Shaden Kamhawi

co-Editor-in-Chief

PLOS Neglected Tropical Diseases

Paul Brindley

co-Editor-in-Chief

PLOS Neglected Tropical Diseases
